# T-type calcium channel inhibition restores sensitivity to MAPK inhibitors in de-differentiated and adaptive melanoma cells

**DOI:** 10.1038/s41416-020-0751-8

**Published:** 2020-02-17

**Authors:** Karol Granados, Laura Hüser, Aniello Federico, Sachindra Sachindra, Gretchen Wolff, Thomas Hielscher, Daniel Novak, Verónica Madrigal-Gamboa, Qian Sun, Marlene Vierthaler, Lionel Larribère, Viktor Umansky, Jochen Utikal

**Affiliations:** 1 Skin Cancer Unit, German Cancer Research Center (DKFZ), Heidelberg and Department of Dermatology, Venereology and Allergology, University Medical Center Mannheim, Ruprecht-Karl University of Heidelberg, D-68135 Mannheim, Germany; 2 Department of Biochemistry, School of Medicine, University of Costa Rica (UCR), Rodrigo Facio Campus, San Pedro Montes Oca, San Jose, 2060 Costa Rica; 3 Department of Hepatology and Gastroenterology, Charité - Universitätsmedizin Berlin, Berlin, Germany; 4 Joint Heidelberg-IDC Translational Diabetes Program, Heidelberg University Hospital, Heidelberg, Germany; 5 Division of Biostatistics, German Cancer Research Center (DKFZ), Heidelberg, Germany

**Keywords:** Targeted therapies, Cancer therapeutic resistance

## Abstract

**Background:**

Drug resistance remains as one of the major challenges in melanoma therapy. It is well known that tumour cells undergo phenotypic switching during melanoma progression, increasing melanoma plasticity and resistance to mitogen-activated protein kinase inhibitors (MAPKi).

**Methods:**

We investigated the melanoma phenotype switching using a partial reprogramming model to de-differentiate murine melanoma cells and target melanoma therapy adaptation against MAPKi.

**Results:**

Here, we show that partially reprogrammed cells are a less proliferative and more de-differentiated cell population, expressing a gene signature for stemness and suppressing melanocyte-specific markers. To investigate adaptation to MAPKi, cells were exposed to B-Raf Proto-Oncogene (BRAF) and mitogen-activated protein kinase kinase (MEK) inhibitors. De-differentiated cells became less sensitive to MAPKi, showed increased cell viability and decreased apoptosis. Furthermore, T-type calcium channels expression increased in adaptive murine cells and in human adaptive melanoma cells. Treatment with the calcium channel blocker mibefradil induced cell death, differentiation and susceptibility to MAPKi in vitro and in vivo.

**Conclusion:**

In summary, we show that partial reprogramming of melanoma cells induces de-differentiation and adaptation to MAPKi. Moreover, we postulated a calcium channel blocker such as mibefradil, as a potential candidate to restore sensitivity to MAPKi in adaptive melanoma cells.

## Background

Malignant melanoma is an aggressive type of skin cancer where the survival rates and treatments vary depending on tumour stages. While early stages have a good prognosis, unresectable stage III and IV melanomas are often fatal and therapy resistance is a major challenge. Around 50% of melanoma patients carry mutations in the BRAF gene and around 30% of patients in the NRAS gene resulting in an over-activation of the mitogen-activated protein kinase (MAPK) pathway,^1^ making this signalling cascade one of the most important targets for melanoma therapy.^2,3^

The clinical use of BRAF inhibitors (vemurafenib, dabrafenib, encorafenib), MEK inhibitors (trametinib, cobimetinib, binimetinib) or their combinations significantly increase progression-free and overall survival of patients.^3,4^ Unfortunately, most patients develop resistance to these inhibitors soon after the start of therapy^5,6^ because of different factors including tumour heterogeneity and plasticity.^7^

The high cellular heterogeneity seen in melanomas is partially due to a degree of phenotypic plasticity. Melanoma cells switch between proliferative/differentiated and invasive/de-differentiated phenotypes during metastasis progression, mimicking the epithelial-to-mesenchymal transition, which facilitates invasion to secondary tumour sites.^8–10^ Indeed, induction of phenotype switching towards a de-differentiated state is likely one of the most common mechanisms underlying the development of resistance to therapies in melanoma patients.^9^

Drug resistance in melanoma has been classified as intrinsic, adaptive or acquired, depending on whether the resistance is present already before treatment starts or it develops within hours or late upon treatment.^2,11^ Several mechanisms have been reported to promote resistance in melanoma, including reactivation of extracellular-signal regulated kinases (ERK) signalling or activation of alternative pathways.^2,5^

Identification of new targets that enhance efficacy of current cancer treatments and prevent development of resistance is essential. T-type calcium channels have been suggested as therapeutic targets in different types of cancer,^12–14^ including melanoma.^15^ These calcium channels play a role in proliferation, survival, regulation of cell cycle and differentiation.^16,17^ Blockage of T-type calcium channels have shown promising results by inducing cytostatic effects on cancer cells that are resistant to standard anti-cancer therapies;^18,19^ however, the use of T-type calcium channels inhibitors in MAPKi-adaptive melanomas, have not been yet described.

Here, we use partial reprogramming,^20^ which represents an intermediate phase of cell fate reversion before fully pluripotency is attained, to investigate de-differentiation and drug adaptation in melanoma. We have found that inhibition of T-type calcium channels in adaptive cells induces differentiation, cell death in vitro and restores sensitisation to MAPKi in vivo. Therefore, we propose use of the calcium channel blocker mibefradil as a novel treatment strategy to overcome adaptation in melanoma.

## Methods

### Cell culture

C790 (*Nras* mutant), 4434 (*Braf*^V600E^ mutant) and 5555 (*Braf*^V600E^ mutant) mouse melanoma cells were kindly provided from Professor Richard Marais (University of Manchester, UK). Human melanoma cell lines A375, HT144 and SK-MEL-28 were obtained from ATCC (Virginia, USA). A375, HT144 and SK-MEL-28 are malignant melanoma cell lines.

All human and mouse melanoma cells were maintained in DMEM medium (Gibco, Thermo Fisher Scientific, USA) supplemented with 10% FCS, 1% nonessential amino acids, 0.75% 2-Mercaptoethanol, 100 units/mL penicillin and 100 μg/mL streptomycin. All cell lines were routinely tested and free of mycoplasma contamination. Reprogramming experiments were performed using KnockOut™ DMEM medium (Gibco, Thermo Fisher Scientific, USA) supplemented with 10% ES-FCS, 1% glutamine, 1% penicillin/streptomycin, 1% nonessential amino acids, 0.75% 2-Mercaptoethanol and 100 U/ ml mLIF. Human melanoma cells were authenticated using the multiplex human cell line authentication test (MCA) (www.multiplexion.de). Mouse melanoma cells were authenticated using the CellCheck Plus-mouse test (www.idexxbioanalytics.eu/).

### Partial reprogramming

Partial reprogramming model was established using mouse melanoma cells C790, 4434 and 5555 as reported previously.^20^ Briefly, cells were transduced with a doxycycline-inducible polycistronic lentiviral vector encoding *Oct4*, *Sox2*, *Klf4* and blasticidin resistance gene, in combination with a lentiviral vector pLU-EF1aL-rtTA3-iCherry (The Wistar Institute, Pennsylvania, USA) encoding for the constitutively active M2 coupled to mCherry. After transduction cells were maintained in reprogramming medium, containing doxycycline (1 μg/ mL) to induce transgene expression. At day 3, cells were selected with blasticidin (8–10 μg/mL) and maintained in culture for twenty days. Pluripotency features were evaluated over reprogramming timeline.

### Lentiviral vectors

HEK293T cells were cultured in DMEM supplemented with 10% FCS, 1% nonessential amino acids, 0.75% mercaptoethanol, and 100 units/mL penicillin and 100 μg/mL streptomycin. For lentiviral production, HEK293T cells were transfected using X-tremeGENE 9 DNA Transfection Reagent (Roche, Basel, Switzerland) according to the manufacturer’s instructions. For the knockdown experiments, target cells were infected with control vector (sh-Scramble) and vector encoding CACNA1H shRNA (TRCN0000044212; Sigma–Aldrich, USA), following the same protocol.

### Inhibitors

The following inhibitors were evaluated: GSK1120212 (Selleckchem, Germany), PLX4032 (Selleckchem, Germany), lomerizine Dihydrichloride (Sigma–Aldrich, USA), mibefradil dihydrochloride hydrates (Sigma–Aldrich, USA), NNC 55–0396 hydrate (Sigma–Aldrich, USA). All drugs were dissolved in DMSO, aliquoted and stored according to the manufacturer’s guidelines.

### Quantitative real-time PCR

Total RNA was isolated using RNeasy kit (Qiagen), DNase I digestion to remove genomic DNA was performed. cDNA was synthesised using cDNA Reverse Transcription kit (Thermo Fisher Scientific, Massachusetts, USA). Quantitative RT-PCR was performed using Applied Biosystems 7500 Real-Time PCR Systems and SYBR Green PCR master mix (Thermo Fisher Scientific, Massachusetts, USA). All reactions were performed at least in triplicates and the amplification signal from the target gene was normalised to GAPDH. PCR conditions were: 50 °C 2 min, 95 °C 10 min, 40 × cycles of 95 °C 15 s, 60 °C 1 min and 72 °C 7 s. The list of primers is shown in Supplementary Table S1.

### Western blot

Total protein lysates were collected using RIPA buffer in presence of phosphatase inhibitors. 20–30 µg of lysates were run on SDS-PAGE gels and transferred to PVDF membranes. Membranes were probed with primary antibodies (1: 1 000 dilutions) overnight at 4 °C and incubated with secondary antibodies (1: 10,000 dilution) for 1 h at room temperature. Chemiluminescence reagents were used. Antibodies used were GAPDH, ERK, p-ERK, SOX2, MITF, Caspase-3 (Cell Signaling Technology, Massachusetts, USA) and CACNA1H (Santa Cruz Biotechnologies, California, USA).

### Cell viability assay

Cells were seeded in triplicates at a density of 1 × 10^3^–1 × 10^5^ cells into 96-well plates. Treatments were tested using a range of concentrations between 0.0001 μM and 10 μM, during 24, 48 and/or 72 h. Cell viability was determined using alamar blue solution (Invitrogen, Thermo Fisher Scientific, USA). After incubation at 37 °C for 4 h, fluorescence emission was measured at 590 nm using a microplate reader (Tecan, Switzerland).

### EdU incorporation assay

Cell proliferation was determined with Click-iT EdU Cell Proliferation Kit according to the manufacturer’s instructions (Invitrogen, Thermo Fisher Scientific, USA). Briefly, EdU (10 μM) was added to the medium for 2 h. Cells were harvested, washed and fixed for 15 min at room temperature in the dark. After fixation cells were washed and stained with Click-iT™ Plus reaction cocktail containing Alexa Fluor™ 647, for 30 min in the dark. Finally, cells were treated with Ribonuclease A, stained with propidium iodide (PI) (50 μg/mL) and analysed by flow cytometry. Data were analysed with FlowJo10x Software.

### Apoptosis assay

Detection of apoptosis was performed using FITC-annexin V apoptosis detection Kit I (RUO) (BD Biosciences, New Jersey, USA). After treatment, supernatants were collected, cells were washed twice with PBS and resuspended in Binding Buffer (1 × ) at a concentration of 1 × 10^6^ cells/mL. Then, cell suspension (100 μL) was stained with FITC-annexin V and propidium iodide (PI). Cells were incubated for 15 min at room temperature in the dark. Then, 400 μL of Binding Buffer (1×) was added to each tube and then analysed by flow cytometry. Doxorubicin (0.003 μM) was used to induce apoptosis as a positive control. Data were analysed using the FlowJo10X Software.

### Clonogenic assay

Colony formation assay was performed following the method described by Franken and co-workers.^21^ Briefly, cells were seeded in 6-well plates for 24 h, after cells were attached, treatments were added for another 24 h. Treatments were withdrawn and fresh medium was added to cells every 3–5 days. After 10–15 days, colonies were fixed and stained with crystal violet staining solution (0.5%) dissolved in methanol. Colony area was calculated using ImageJ software.

### Invasion assay

To assess invasion, Corning™ BioCoat™ Tumor Invasion 24-Multiwell Plates containing FluoroBlok^TM^ PET membrane were used (Corning^®^, New York, USA). First, plates were rehydrated for 2 h at 37 °C. Then, 500 μL of cell suspension (3 × 10^4^ cells/mL) was added to the apical chambers and 750 μL of chemoattractant (10% FCS in DMEM) to the basal chambers. Plate was incubated for 22 h at 37 °C and 5% CO_2_. After incubation, Calcein (4 µg/mL) was added to a second 24-well plate and incubated during 1 h at 37 °C and 5% CO_2_. Finally, Calcein solution was removed before reading fluorescence of invading cells at 517 nm, using a microplate reader (Tecan, Switzerland). Data were expressed as relative fluorescent units (RFU) of invaded cells.

### In vivo experiments and drug administration

In vivo experiments were assessed using NSG xenograft mouse model. Four to six-week-old female mice were injected subcutaneously (SC) with 100 μL PBS containing 5 × 10^6^ HT144 cells or 1 × 10^6^ A375 cells. Tumour-bearing mice (100–300 mm^3^) were randomised (*n* = 40) and treated in one of the following treatments: (1) mibefradil (0.25 mg/mL) in drinking water for 5 days; (2) vemurafenib (100 mg/kg) dissolved in HPMC solution (0.5%) by oral gavage once a day for 5 days; (3) sequential treatment: vemurafenib by oral gavage once a day for 5 days and mibefradil in drinking water on the next 5 days and (4) vehicle: 0.5% HPMC solution by oral gavage once a day for 5 days. Animals were treated in 2 cycles. Tumour volume, animal weight (18–20 Kg) and welfare were monitored daily. Tumour measurements were conducted using a calliper and tumour volume was calculated using the following formula = (length × width^2^) × 0.5. Animals were killed by cervical dislocation once the tumour volume reached 1000 mm^3^. Drug administration or euthanasia did not require the use of anaesthesia. Animals were maintained in the animal house facility under optimal conditions and with food and water ad libitum. All animal experiments were performed following according to procedures approved by the German authorities (Animal grant approval number: 35–9185.5/G-208/18).

### Microarray and bioinformatic analysis

Total RNA was isolated with RNeasy Mini Kit (Qiagen, Germantown, MD, USA) according to the manufacturer’s instructions. The concentration and purity of total RNA were determined by spectrophotometry. RNA was labelled and hybridised to GeneChip^®^ Mouse Genome 430 2.0 Array (Affymetrix, Santa Clara, CA, USA) by DKFZ Genomics and Proteomics Core Facility. Fold change and p-value of differentially expressed genes between + dox vs − dox cells were derived from the CEL files, RMA normalisation method was done using Chipster Software. Additional samples were hybridised to Illumina MouseRef-8 v2.0 Expression BeadChips by DKFZ Genomics and Proteomics Core Facility. Fold change and p-value of differentially expressed genes were derived from Chipster Software. Further analyses were made using Ingenuity Pathway Analysis (IPA) (Ingenuity^®^ Systems, CA, USA, www.ingenuity.com). IPA software was used to identify upstream pathways, regulators and predictors. Microarray data can be downloaded with the GEO accession number: GSE122399, GSE122402 and GSE122763.

RNA-Seq data for 459 melanoma patients with follow-up information was downloaded from the TCGA-SKCM project (https://portal.gdc.cancer.gov/projects/TCGA-SKCM), including 357 metastatic samples and 102 primary tumour samples. Recursive partitioning^22^ based on tissue type and log2-transformed FPKM expression values for CACNA1H was performed to identify risk groups for overall survival. P-value was adjusted for multiple testing using Bonferroni correction. Software R 3.5 including add-on packages TCGAbiolinks^23^ and partykit were used for analysis.

### Statistical analysis

Data are expressed as the means ± SEM from three or more independent experiments. Differences were analysed by one-way ANOVA followed by post-hoc test Bonferroni or Tukey. Data analyses were performed with GraphPad Prism (GraphPad Software, Inc.). *P* < 0.05 was considered statistically significant. Log-rank test was used to compare survival curves. Tumour growth curves were compared between treatments by fitting a linear mixed model for tumour volume with predictor time, treatment and interaction between time and treatment as fixed effects, and random intercept and slope effect for each mouse. The interaction term was tested to compare the growth rate relative to the vehicle group. *P*-values were adjusted for multiple comparisons using Holm correction. Software R 3.5 was used for analysis.

## Results

### Partial reprogramming of melanoma cells induces de-differentiation and an invasive phenotype in murine melanoma cells

**Fig. 1:**
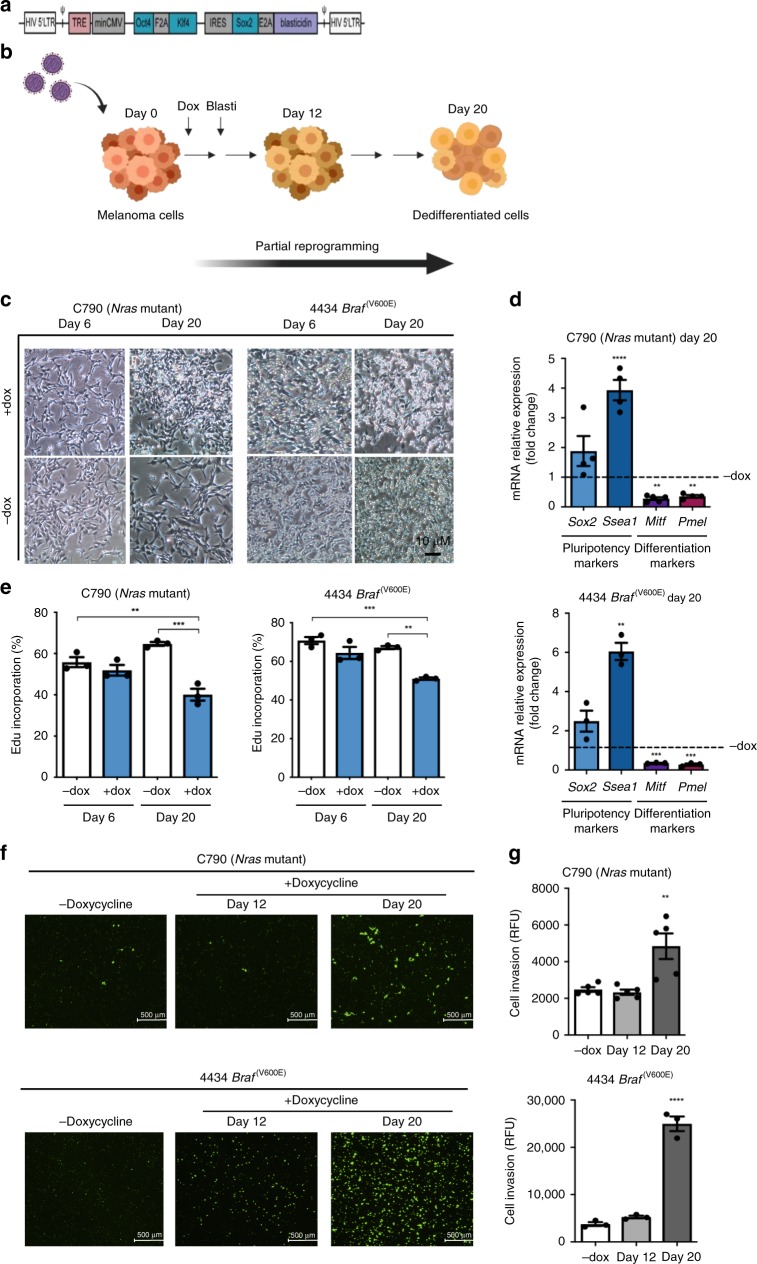
Partial reprogramming of murine cells leads to a less proliferative and more de-differentiated cell population. **a** Structure of lentiviral vector used to reprogram cells. Cells were infected with M2mCherry vector plus an expression vector carrying murine *Oct4*, *Klf4* and *Sox2* under the control of a tetracycline-responsible element (TRE) and blasticidin-resistant gene. Cells not induced with doxycycline but expressing both vectors were used as a control. **b** Schematic representation of partial reprogramming method. After infection, cells were treated with doxycycline and selected with blasticidin at day 3. Medium without doxycycline was used for control cells during all experiments. **c** Representative images of morphological changes during partial reprogramming. Scale bars represent 10 μm. **d** Real-Time qPCR analysis for stemness markers *Sox2* and *Ssea-1*, as well as for melanocytic lineage differentiation markers *Mitf* and *Pmel*, for C790 and 4434 at day 20 of reprogramming. Data were normalised using control cells (“− dox”) as reference and *Gapdh* as housekeeping gene. **e** Cell proliferation measured by EdU incorporation. Proliferation was analysed at different time points over partial reprogramming as indicated. After incubation with EdU cells were analysed by flow cytometry. Percentage of EdU-positive cells is shown. **f** Representative images of fluorescence microscopy for cell invasion assay in C790 and 4434 cells. Scale bars represent 500 μm. **g** Cell invasion assessed by the FluoroBlok invasion assay. Invasion was evaluated at different time points during partial reprogramming. After 20 h incubation, invading cells were labelled and relative fluorescence units were obtained (RFUs = relative fluorescence units). All data are represented as mean ± SEM of three or more independent experiments. Statistical analyses were performed with One-Way ANOVA and post-hoc test; **p < 0.01, ***p < 0.001 and ****p < 0.0001.

Melanoma progression has been described as a step-wise transformation of melanocytes to malignant melanoma triggered by a phenotypic switch toward a more aggressive status.^24^ To determine whether our in vitro model of cellular reprogramming could simulate features of melanoma phenotype switching and the intermediate stages during melanoma progression, we partially reprogrammed *Nras*-mutated C790 and *Braf*-mutated 4434 murine melanoma cells for up to 20 days using a lentiviral vector carrying *Oct4*, *Klf4* and *Sox2* genes (Fig. 1a, b*)*.

Morphological changes were observed during partial reprogramming (Fig. 1c). Additionally, analysis of markers of stemness and melanocytic lineage differentiation showed that *Mitf* and *Pmel* expression was significantly decreased while expression of *Sox2* and *Ssea-1* increased, confirming that cells were de-differentiated (Fig. 1d *and* Supplementary Fig. S1a). RNA array data further supported the partial reprogramming of C790 and 4434 murine cells (Supplementary Fig. S1b, c). Moreover, C790 and 4434 parental cells were treated with doxycycline for 20 days to evaluate whether doxycycline has an effect on the expression of stemness and melanocytic lineage differentiation markers (Supplementary Fig. S1d, e).

During melanoma phenotype switching, cells transit to a more invasive, slow-cycling state.^25^ EdU incorporation and Transwell invasion assays revealed a significant reduction in proliferation of reprogrammed cells (Fig. 1e) and an increased number of invading cells (Fig. 1f, g); confirming a phenotypic switch towards a de-differentiated, aggressive cell type.

Additional *Braf*-mutated murine melanoma cells (5555) were partially reprogrammed for 20 days. Expression of markers of stemness was enhanced while expression of melanocytic lineage differentiation was decreased (Supplementary Fig. S2a). Phenotypic switch was also confirmed with a reduction in proliferation (Supplementary Fig. S2b) and with a more invasive phenotype (Supplementary Fig. S2c, d).

### Partially reprogrammed melanoma cells adapt to MAPK inhibitors

**Fig. 2:**
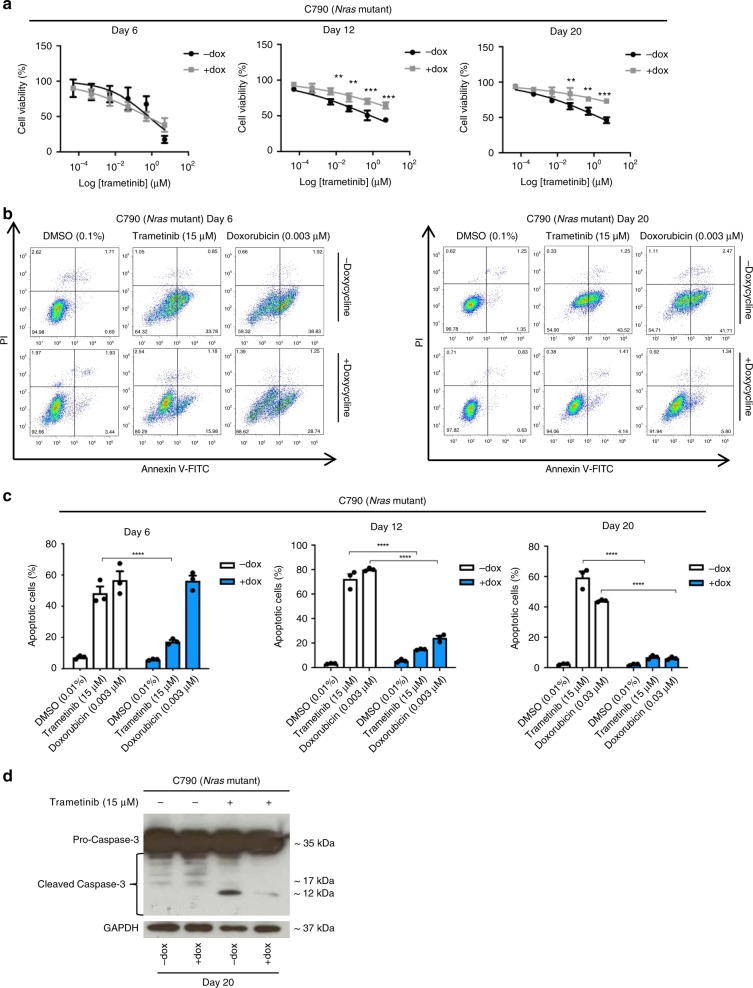
Partially reprogrammed cells adapt to MAPK inhibitors (MAPKi). **a** Cell viability performed using alamar blue assay. *Nras-*mutant cells (C790) were treated with trametinib (10–0.0001 μM) for 72 h, after 3 h incubation with alamar blue, fluorescence emission (read at 590 nm) was obtained. Cell viability was evaluated at days 6, 12 and 20 of reprogramming of C790 cells. Cytotoxicity curves represent as mean ± SEM (*n* = 6). **b** Representative scatter plots of PI (y-axis) vs. annexin V (x-axis) for day 6 and 20 of partial reprogramming. Early apoptotic cells are shown in the lower right quadrant and late apoptotic cells are shown in the upper right quadrant. **c** Apoptosis after staining with FITC-Annexin V/PI. Cells were treated with trametinib (15 μM) for 72 h and with doxorubicin (0.003 μM) to induce apoptosis (positive control). Percentage of apoptotic cells (early and late apoptosis) is shown as mean ± SEM (*n* = 3). **d** Western blot analysis. Whole cell lysates were immunoblotted with GAPDH and caspase-3 antibodies at day 20 of reprogramming after treatment with trametinib. GAPDH loading control is identical in western blots of Figs. 2d and 3a at day 20, since it was analysed on the same blot. Statistical analyses were performed with One-Way ANOVA and post-hoc test; **p < 0.01, ***p < 0.001 and ****p < 0.0001.

De-differentiation of melanoma tumour cells has been associated with development of resistance to therapy.^26,27^ We evaluated the effect of BRAF and MEK inhibitors on the cell viability and cell death of C790 and 4434 partially reprogrammed cells. Cell viability was measured after 72 h of treatment with trametinib, vemurafenib or the combination of trametinib and vemurafenib. Cell viability increased in partially reprogrammed cells (“ + dox”) throughout the days of reprogramming compared to control cells (“− dox”). Treatment with trametinib alone revealed that C790 control cells are more sensitive (mean IC50 = 2.09 ± 0.48 μM) compared to C790 reprogrammed cells at day 20 (mean IC50 > 10 μM) (Fig. 2a). Combination of vemurafenib and trametinib in 4434 reprogrammed cells also showed a higher IC50 value at day 20 (mean IC50 = 4.38 ± 0.03 μM) compared to 4434 control cells (mean IC50 = 0.0029 ± 0.0010 μM) (Supplementary Fig. S3a). Mean of IC50 values for all treatments were presented in Supplementary Tables S2 and S3.

We further evaluated whether cell death is also affected in partially reprogrammed cells after treatment with MAPKi by measuring apoptosis with FITC-annexin V/PI staining. After treatment with trametinib (15 μM), we found that the percentage of apoptotic cells was significantly reduced at day 12 and 20 of reprogramming in C790 de-differentiated cells ( + dox) compared to control cells (−dox). Percentage of apoptosis was also reduced in reprogrammed cells after treatment with doxorubicin (0.003 μM) (Fig. 2b, c). Similar results were obtained at day 20 for 4434 reprogrammed cells, treated with trametinib (8 μM), vemurafenib (15 μM) or the combination of both at a ratio 1:1 (8 μM) (Supplementary Fig. S3b, c). In addition, activation of caspase-3 was suppressed in reprogrammed cells after treatment with MAPKi compared to control cells (Fig. 2d and Supplementary Fig. S3d*)*. We also confirmed a decrease in cell death (Supplementary Fig. S2e, f) and activation of caspase-3 (Supplementary Fig. S2g) in 5555 partially reprogrammed cells at day 20 after treatment with the combination of trametinib and vemurafenib. All these findings support that partially reprogrammed cells become less sensitive to MAPKi.

### MAPKi-adaptive cells overexpress T-type calcium channels

**Fig. 3:**
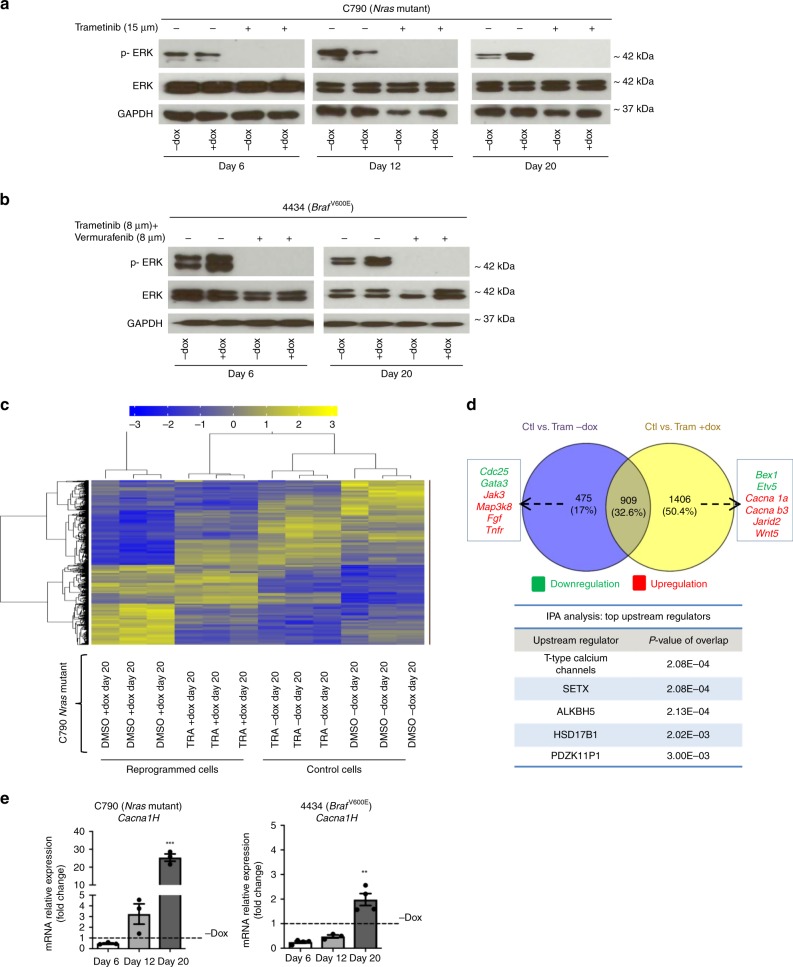
Expression analysis of MAPKi-adaptive cells shows upregulation of T-Type calcium channels. **a**, **b** Western blot analysis. Whole cell lysates were immunoblotted with GAPDH, ERK and P-ERK antibodies in C790 and 4434 melanoma cells. GAPDH loading control is identical in western blots of Figs. 2d and 3a at day 20, since it was analysed on the same blot **c** Differential gene expression analysis. Heat map of microarray data showing hierarchical clustering of 2700 differentially expressed genes in partially reprogrammed cells C790 after 72 h treatment with DMSO (control) and trametinib; control (“−dox”) and reprogrammed cells (“ + dox”) were evaluated both at day 20. Blue or yellow colours indicate differentially up- or downregulated genes, respectively (FC > 2-fold). **d** Analysis of gene expression data by a Venn diagram showing the analysis of treatments between control cells and reprogrammed cells (2700 genes). The blue circle (474 genes) indicates the number of genes exclusively expressed in control vs. trametinib (“−dox”); yellow circle (1406 genes) indicates the number of genes exclusively expressed in control vs. trametinib (“ + dox”). Some of the deregulated genes are listed; green and red colours indicate differentially up- or downregulated genes, respectively. Moreover, results from IPA analysis of upstream regulators from C790 partially reprogrammed cells at day 20 after treatment with trametinib are listed. **e** Real-Time qPCR analysis for Cacna1h at different days of reprogramming for both C790 and 4434 cells. Data were normalised using control cells as reference and GAPDH as housekeeping gene. All data are represented as mean ± SEM of three or more independent experiments. Statistical analyses were performed with One-Way ANOVA and post-hoc test; **p < 0.01 and ***p < 0.001.

Since partially reprogrammed cells were less sensitive to MAPKi we wondered whether ERK activation was also affected following treatment with trametinib or with the combination of trametinib and vemurafenib. Western blot analysis revealed that phosphorylation of ERK is impaired in both C790 and 4434 partially reprogrammed cells and control cells (Fig. 3a, b), indicating that MAPK pathway inhibition is independent from the de-differentiation status of the cells.

We further investigated the mechanisms of adaptation in de-differentiated cells using gene expression array analysis for C790 reprogrammed cells at day 20 after the treatment with trametinib (Fig. 3c). The dendrogram showed some of the genes differentially expressed in reprogrammed cells compared to control cells, +dox vs −dox. Moreover, IPA software was used to analyse upstream regulators that might be involved in regulation of drug sensitivity after partial reprograming of melanoma cells (Fig. 3d). Interestingly, T-type calcium channels were predicted to be one of the significant upstream regulators in adaptive cells, suggesting them as a target to alleviate therapy adaptation in melanoma. Moreover, analysis from TCGA-SKCM database showed that melanoma patients with high expression of CACNA1H have a worse survival outcome compared with those with low CACNA1H expression (*p* = 0.029) (Supplementary Fig. S4a) Furthermore, we confirmed that *Cacna1h* was highly expressed in both C790 and 4434 partially reprogrammed cells (Fig. 3e and Supplementary Fig. S4b), highlighting the importance of calcium in adaptation to MAPKi.

Based on these data, we were interested to further explore the effect of T-type calcium channels in de-differentiated and adaptive melanoma cells. We speculated that adaptation to MAPKi could be regulated by an alternative pathway, in which T-type calcium channels may be involved.

### Inhibition of T-type calcium channels increases drug-sensitivity and differentiation in murine MAPKi-adaptive melanoma cells

**Fig. 4:**
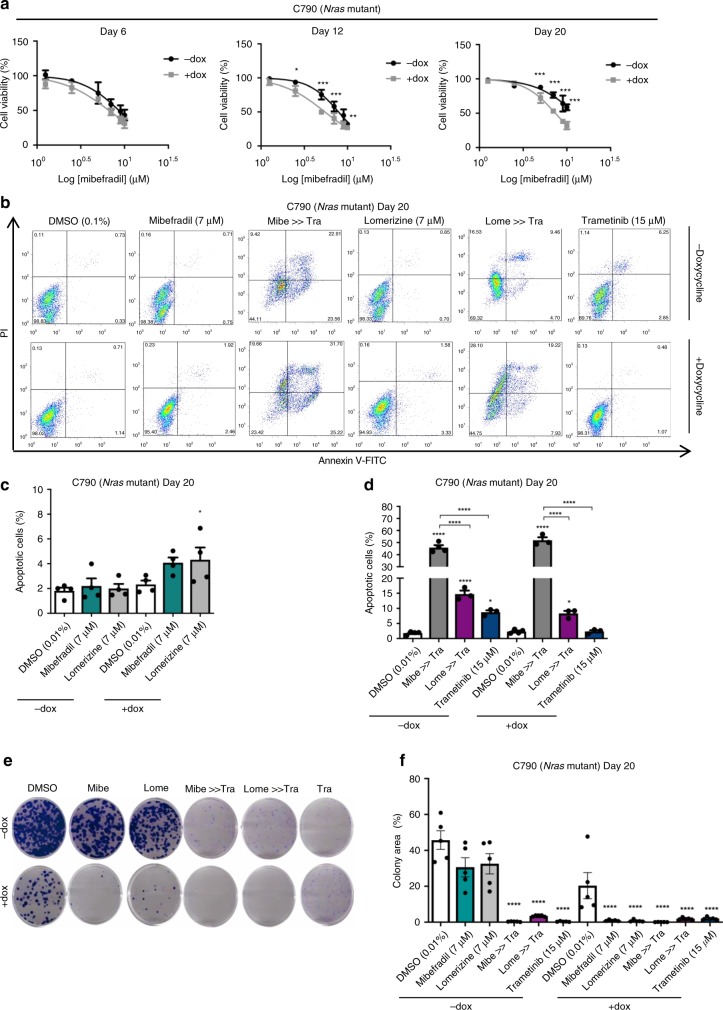
Inhibition of calcium channels increases sensitivity to MAPK inhibitors in partially reprogrammed cells. **a** Cell viability was performed using alamar blue assay. Partially reprogrammed cells C790 were treated with mibefradil (10–1.25 μM) for 24 h. After 3 h incubation with alamar blue, fluorescence (at 590 nm) was obtained. Cell viability was evaluated at days 6, 12 and 20 of reprogramming. **b** Representative scatter plots of PI (*y*-axis) vs. annexin V (*x*-axis) for day 20 of partial reprogramming. Early apoptotic cells are shown in the lower right quadrant and late apoptotic cells are shown in the upper right quadrant. Mibe: mibefradil (7 μM). Lome: lomerizine (7 μM). Mibe (Lome) »Tra: sequential treatment with mibefradil (lomerizine) 24 h, followed by trametinib (15 μM) for another 24 h. **c** Apoptosis analysis using annexinV/PI staining. Cells were treated with mibefradil (7 μM) and lomerizine (7 μM) for 24 h. Percentage of apoptotic cells (early and late apoptosis) is shown as mean ± SEM (*n* = 3). **d**. Apoptosis analysis using sequential treatment with calcium channel blockers and MAPKi. Percentage of apoptotic cells (early and late apoptosis) is shown as mean ± SEM (*n* = 3). **e** Clonogenic assay of partially reprogrammed cells C790 treated with DMSO (0.01%), mibefradil (7 μM), lomerizine (7 μM), Mibe (Lome) »Tra: sequential treatment with mibefradil (lomerizine), and trametinib (15 μM) for 24 h. Representative images of wells stained with crystal violet are shown. **f** Percentage of colony area for all treatments is shown as mean ± SEM (*n* = 5) for partially reprogrammed cells C790. All data are represented as mean ± SEM of three or more independent experiments. Statistical analyses were performed with One-Way ANOVA and post-hoc test; *p < 0.05, **p < 0.01 and ***p < 0.001.

To determine whether the inhibition of T-type calcium channels could induce cell death in de-differentiated melanoma cells, C790 and 4434 reprogrammed cells were treated with mibefradil and lomerizine for 24 h. Cell viability showed no significant differences in the IC50 values of mibefradil in partially reprogrammed cells (“ + dox”), compared to control cells (“− dox”) (Fig. 4a, Supplementary Fig. S5a). De-differentiated cells were slightly sensitive to mibefradil (C790 mean IC50 = 5.9 ± 0.74 μM; 4434 mean IC50 = 8.87 ± 0.25 μM) compared to control cells (C790 mean IC50 = 6.44 ± 0.66 μM; 4434 mean IC50 = 7.82 ± 0.96 μM). Similar results were observed with lomerizine (Supplementary Tables S2 and S4). Moreover, a more selective T-type calcium channels inhibitor (NNC 55–0396) was also evaluated. Results showed that IC50 values of NNC 55–0396 were significantly different between partially reprogrammed cells (“ + dox”), compared to control cells (“− dox”) (Supplementary Tables S5 and S6).

Furthermore, single treatments with mibefradil and lomerizine slightly increased the percentage of apoptotic cells after 24 h in both C790 (Fig. 4b, c) and 4434 partially reprogrammed cells (Supplementary Fig. S5b, c). Together, these data indicated that inhibition of T-type calcium channels has the potential to promote apoptosis and affect cell viability in de-differentiated and adaptive cells.

Considering these results and the high expression of T-type calcium channel in de-differentiated cells (Fig. 3e and Supplementary Fig. S4b), we hypothesised that these channels could be potential candidates to re-sensitise adaptive cells to MAPKi. Therefore, we evaluated drug sensitisation by treating reprogramed cells with mibefradil or lomerizine for 24 h followed by treatment with MAPKi for another 24 h (sequential treatment). The percentage of apoptotic cells was significantly increased in partially reprogrammed cells after the sequential treatment (Fig. 4d and Supplementary Fig. S5d), and the capacity of colony formation was significantly reduced in C790 partially reprogrammed cells after sequential treatment, (Fig. 4e, f), suggesting that previous sensitisation with mibefradil increased cell death and decreased the capacity of unlimited cell reproduction after treatment with MAPKi. Similar results were obtained in 4434 partially reprogrammed cells (Supplementary Fig. S5e, f).

Since calcium participates as a second messenger in a wide variety of cellular mechanisms, identifying how calcium regulates cellular capacity for self-renewal is challenging. We speculated that T-type calcium channels affect the pluripotency status of reprogrammed cells. Treatment with mibefradil and lomerizine in C790 and 4434 partially reprogrammed cells revealed that the expression of stemness markers *Sox2*, *Ssea-1* and *CD271* were significantly reduced (Supplementary Fig. S6a, b), leading into a more differentiated phenotype. Together these data suggest that the modulation of calcium influx improved responses to MAPKi-adaptive cells by promoting cell death and differentiation.

### Calcium channel antagonists sensitise human BRAF-adaptive melanoma cells

**Fig. 5:**
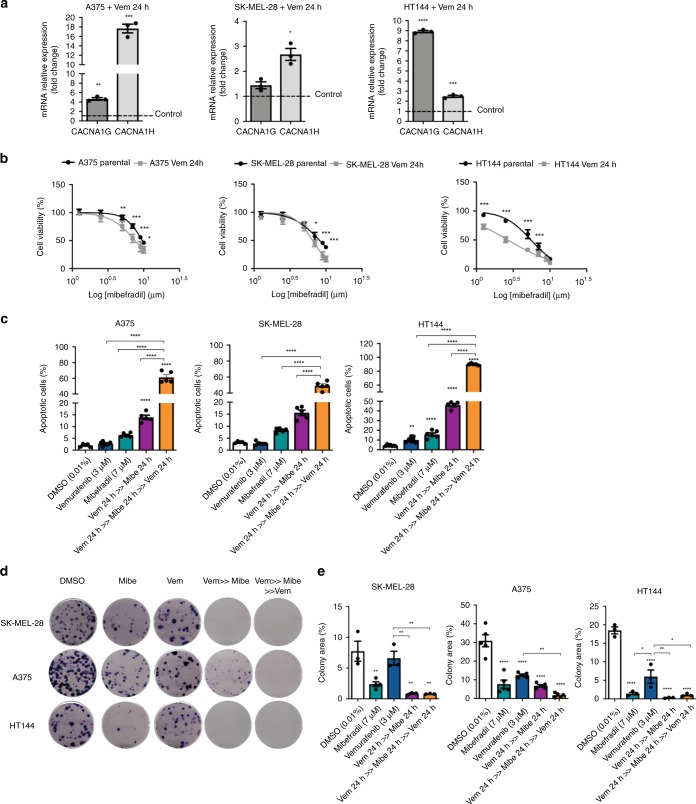
Mibefradil increases vulnerability to MAPK inhibitors in human BRAF- adaptive melanoma cells. **a** Gene expression analysis of CACNA1G and CACNA1H in human melanoma cell lines A375, SK-MEL-28 and HT144 upon 24 h treatment with vemurafenib (3 μM) (adaptation). Data were normalised using control cells as reference and 18 S as housekeeping gene. **b** Human melanoma cells were treated with mibefradil and lomerizine (10, 9, 7, 5, 2.5 and 1.25 μM) for 24 h (only mibefradil treatment is shown). After 3 h of incubation with alamar blue, fluorescence (590 nm) was obtained. **c** Apoptosis analysis of human melanoma, cells were treated with vemurafenib (3 μM) for 24 h, followed by mibefradil (7 μM) for 24 h; after this period cells were re-treated with vemurafenib (3 μM) for another 24 h (“Vem 24 h » Mibe 24 h » Vem 24 h”). Cells were stained and analysed by flow cytometry. Percentage of apoptotic cells (early and late apoptosis) is shown as mean ± SEM (*n* = 5). **d** Clonogenic assay of human cells A375, SK-MEL-28 and HT144 treated for 24 h with DMSO (0.01%), mibefradil (7 μM), vemurafenib (3 μM) and sequential treatment (Vem 24 h » Mibe 24 h » Vem 24 h). Representative images of wells stained with crystal violet are shown. **e** Percentage of colony area for all treatments is shown as mean ± SEM (*n* = 3) in all human melanoma cell lines. All data are represented as mean ± SEM of three or more independent experiments. Statistical analyses were performed with One-Way ANOVA and post-hoc test; *p < 0.05, **p < 0.01, ***p < 0.001 and ****p < 0.0001.

To establish the role T-type calcium channels play in adaptive human melanoma cells, we used BRAFi-adaptive melanoma cells A375, SK-MEL-28 and HT144 treated for 24 h with vemurafenib (3 µM). We confirmed that human vemurafenib-adaptive melanoma cells increased expression of T-type calcium channels genes, CACNA1G and CACNA1H compared to parental cells (Fig. 5a).

To determine if the increment in the expression of calcium channels was associated with a more de-differentiated and adaptive phenotype, similar to reprogrammed murine cells, we tested the effect of mibefradil on cell viability, cell death and colony formation ability in human BRAFi-adaptive cells. After 24 h treatment, mibefradil reduced viability in adaptive cells (A375 mean IC50 = 7.43 ± 0.80 μM; SK-MEL-28 mean IC50 = 6.5 ± 0.30 μM; HT144 mean IC50 = 2.65 ± 0.14 μM) compared to parental cells (A375 mean IC50 = 9.63 ± 0.35 μM; SK-MEL-28 mean IC50 = 8.17 ± 0.40 μM; HT144 mean IC50 = 5.5 ± 0.41 μM) (Fig. 5b *and* Supplementary Table S7). Treatment with lomerizine did not show a significant effect on the cell viability (Supplementary Fig. S7a) but, treatment with NNC 55–0396 did show significant differences between adaptive cells and parental cells (Supplementary Fig. S8a and Supplementary Table S8). Moreover, single treatment with mibefradil had no effect on apoptosis of parental cells but increased cell death in all adaptive cells (“Vem24h » Mibe24h”) and increased sensitivity to vemurafenib (“Vem24h » Mibe24h » Vem24h”) (Fig. 5c *and* Supplementary Fig. S7b). In addition, colony formation capacity was completely reduced in all adaptive cells after sequential treatment (“Vem24h » Mibe24h » Vem24h”) in comparison with single treatment with vemurafenib or DMSO (Fig. 5d, e). Treatment with NNC 55–0396 also increased cell death (Supplementary Fig. S8b) and impaired colony formation capacity (Supplementary Fig. S8c, d) in adaptive cells after sequential treatment (“Vem24h » NNC » Vem24h”).

These results were consistent with observations in partially reprogrammed murine cells and confirmed the possibility of using calcium channels blockers to sensitise adaptive cells to MAPK inhibitors.

In agreement with earlier results, treatment with mibefradil on human adaptive cells suppressed expression of differentiation markers associated with adaptive resistance^28,29^ in both parental and adaptive cells (Supplementary Fig. S9a, b, c). Sequential treatment (“Vem24h » Mibe24h » Vem24h”) showed a strong reduction of SOX2 expression, mRNA and protein levels, in SK-MEL-28 and HT144 adaptive cell lines (Supplementary Fig. S9d), supporting a possible connection between calcium signalling and stemness maintenance.

### Silencing of CACNA1H promotes cell death and differentiation in human BRAF-adaptive melanoma cells

**Fig. 6:**
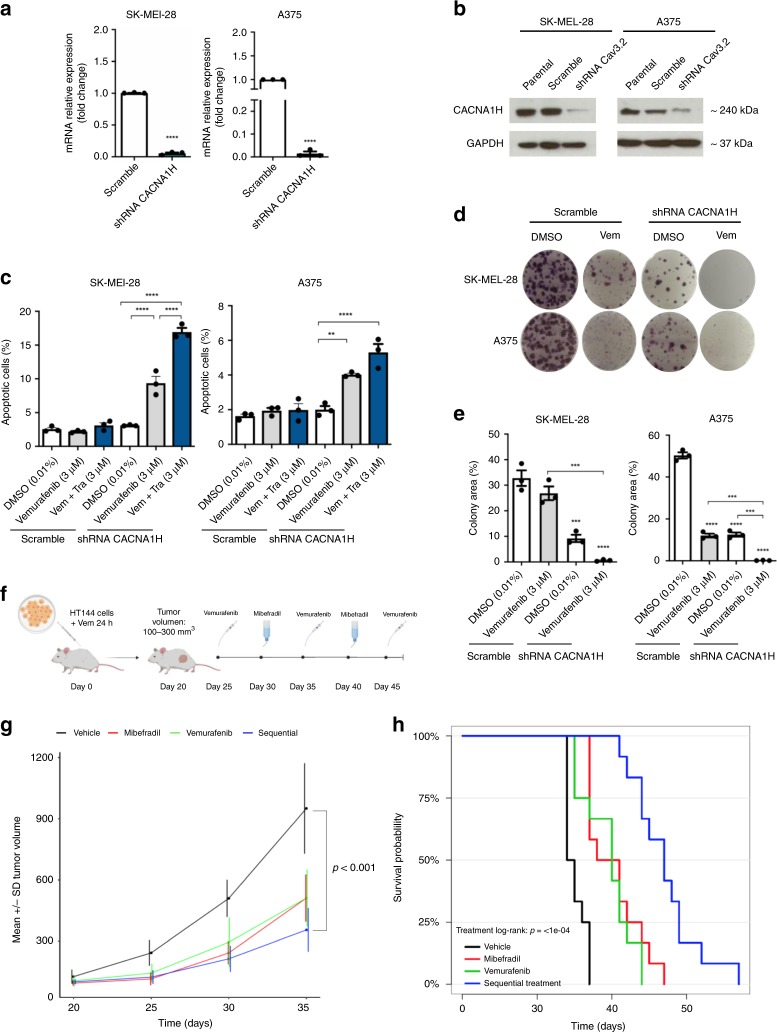
Blockage of CACNA1H induces cell death and differentiation in vitro and tumour growth inhibition in vivo in human BRAFi-adaptive melanoma xenografts. **a** Human melanoma cells A375, SK-MEL-28 and HT144 were transfected with sh- CACNA1H or sh-control (Scramble). After 48 h selection with puromycin (0.8 μg/mL), cells were collected and analysed by qPCR and western blot. Gene expression of CACNA1H confirms the silencing of the gene in cell lines A375 and SK-MEL-28. **b** Western blot analysis. Protein lysates of human melanoma cells were immunoblotted with GAPDH and CACNA1H antibodies. **c** Apoptosis assay of A375 and SK-MEL-28 cell lines. After treatment with sh- CACNA1H, cells were stained with annexinV/PI to analyse apoptosis by flow cytometry. Percentage of apoptotic cells (early and late apoptosis) is shown as mean ± SEM (*n* = 3). **d** Clonogenic assay of human cell lines A375 and SK-MEL-28 transfected with sh- CACNA1H or sh-control (Scramble), treated for 24 h with DMSO (0.01%) and vemurafenib (3 μM). Representative images of wells stained with crystal violet are shown. **e** Percentage of colony area for all treatments is shown as mean ± SEM (*n* = 3) in all human melanoma cell lines. **f** Workflow for in vivo evaluation of sequential treatment (Vem » Mibe » Vem) in adaptive human cells. HT144 cells treated with vemurafenib for 24 h were injected subcutaneously in female NGS mice. Once tumour volume reached 100–300 mm^3^, animals were randomised into four groups (*n* = 10), and treatments were administrated daily as is shown. **g** Effect of sequential treatment in tumour growth in HT144 xenografts, shown as mean and standard deviation of tumour volume over time following treatment initiation. Tumour growth curves were compared based on a linear mixed model with predictors time, treatment and interaction between time and treatments as fixed effects, and random intercept/slope effect. The interaction term was tested to compare the growth rate relative to the vehicle group. P-values are adjusted for multiple testing. **h** Kaplan-Meier curves representing survival of HT144 xenografts mice treated with vehicle (black line), mibefradil (red line), vemurafenib (green line) and sequential treatment (blue line). Survival end point was established when the tumour volume reached 1000 mm^3^. The survival curves were analysed with pairwise treatment comparison using log-rank test with adjustment of p-values for multiple testing. Data are represented as mean ± SEM of three independent experiments. Statistical analyses were performed with One-Way ANOVA and post-hoc test; **p < 0.01, ***p < 0.001 and ****p < 0.0001.

To test whether CACNA1H is responsible for the increase in apoptosis and sensitisation in adaptive cells, we silenced CACNA1H gene in human melanoma cells (A375, SK-MEL-28 and HT144) and analysed the effect on cell death and differentiation after treatment with vemurafenib. CACNA1H knockdown was verified by qPCR and western blot in all cell lines (Fig. 6a, b and Supplementary Fig. S10a, b).

We evaluated apoptosis in CACNA1H knockdown cell lines after 24 h of treatment with vemurafenib or the combination treatment with vemurafenib and trametinib. CACNA1H knockdown increased apoptosis in CACNA1H knockdown cell lines compared to the scramble control (Fig. 6c), thereby confirming that induction of apoptosis in BRAFi-adaptive cells was driven by inhibition of CACNA1H calcium channel. Due to limited efficiency of knockdown (Supplementary Fig. S10b) there was no significant difference in cell death for HT144 CACNA1H knockdown cells (Supplementary Fig. S10c). Moreover, colony formation capacity was affected in CACNA1H knockdown cells lines A375 and SK-MEL-28 after treatment with vemurafenib (Fig. 6d, e), supporting that CACNA1H calcium channel knockdown increased sensitivity of adaptive cells to BRAFi.

To confirm that knockdown of CACNA1H was responsible for changes in differentiation status, we measured SOX2 expression in human melanoma cells. mRNA and protein levels were substantially reduced in both A375 and SK-MEL-28 cells indicating decreased stemness phenotype (Supplementary Fig. S10d, e). Taking together, CACNA1H silencing induced a similar effect on cell death and differentiation as the pharmacological inhibition of calcium channels with mibefradil.

### Sequential treatment using mibefradil and vemurafenib reduces tumour growth and improves survival in HT144 and A375 adaptive xenografts model

To determine the anti-tumour effect of the sequential treatment in vivo, HT144 and A375 xenografts model were used. Mice were inoculated subcutaneously either with HT144 or A375 cells treated for 24 h with vemurafenib. After tumour volume reached 100–300 mm^3^, animals (*n* = 40) were randomised to four different treatment groups. Sequential treatment consists in oral administration of vemurafenib for 5 days, follow by mibefradil for another 5 days. This workflow was repeat until two cycles were completed and finally, vemurafenib was apply for the last 5 days of the experiment (Fig. 6f and Supplementary Fig. S10f). In parallel, single treatments with vemurafenib, mibefradil or vehicle were tested.

Single treatments with vemurafenib (*p* = 0.013) and mibefradil (*p* = 0.025) significantly reduced tumour growth compared to control group (vehicle) while sequential treatment (*p* < 0.001) showed the most significant reduction on tumour volume compared to control mice (vehicle) in HT144 xenografts (Fig. 6g). Moreover, survival data showed a better outcome for mice treated with sequential treatment compared to control group (*p* < 0.001) (Fig. 6h). In addition, single treatments of vemurafenib (*p* = 0.001) and mibefradil (*p* < 0.001) showed a significant difference in survival compared to vehicle group. These results support the use of mibefradil to enhance the anti-tumour effects of vemurafenib in vivo.

In A375 xenografts, single treatment with vemurafenib alone led to a slight reduction in tumour growth compared to control group (vehicle). In contrast, mice treated with mibefradil (*p* = 0.013) or sequential treatment (*p* = 0.019) had a significant reduction in tumour volume compared to control mice (vehicle) (Supplementary Fig. S10g). Considering the survival data, mice treated with sequential treatment showed a better outcome, compared to control group (*p* < 0.001) (Supplementary Fig. S10h), similar to the results obtained in HT144 xenografts. Single treatments of vemurafenib (*p* = 0.053) and mibefradil (*p* = 0.063) did not show significant difference in survival compared to vehicle group.

These in vivo effects are consistent with our previous in vitro data in human adaptive cells and mouse reprogrammed cells, suggesting that the inhibition of T-type calcium channels is a promising strategy to sensitise de-differentiated and adaptive melanoma cells to MAPKi.

## Discussion

Here, we have demonstrated that partial reprogramming of melanoma cells induced a de-differentiated phenotype and increased adaptation against MAPKi. We observed that T-type calcium channels expression increased in MAPKi-adaptive melanoma cells and that inhibition of T-type calcium channels enhanced cell death and differentiation in reprogrammed melanoma cells and in human BRAFi-adaptive melanoma cells. Based on the reduction of tumour growth and the improvement in overall survival after sequential treatment *in vivo*, we suggest that T-type calcium channels are potential targets to eliminate adaptive melanoma cells by restoring sensitivity to MAPKi.

In vitro de-differentiation or reprogramming of melanoma cells has been described previously.^20,30–32^ *Nras* and *Braf*-mutated melanoma cells were partially reprogrammed to study genetic and molecular changes during the intermediate stages of tumour progression. We observed a switch in gene expression of partially reprogrammed cells with increased expression of stemness related genes, reduced levels of melanocytic markers as well as decreased proliferation and increased invasiveness, which are consistent with the classical features of phenotype switching in melanoma.^24,33^ Considering that cellular de-differentiation has been correlated with development of resistance to therapies,^6,33,34^ and that progressive de-differentiation status of melanoma has been observed in patient samples under standard treatments with MAPKi,^26^ we hypothesised that partially reprogrammed melanoma cells would be less sensitive to BRAF or MEK inhibitors. Consistent with other reports where de-differentiation of human melanoma cells conveyed increased adaptation to MAPKi,^31^ our results indicated that partially reprogrammed murine melanoma cells also increased survival after treatment with trametinib, vemurafenib or with their combination.

Several mechanisms have been implicated^2,5,35,36^ in acquired, adaptive and intrinsic resistance in melanoma, although most have included reactivation of ERK signalling with some exceptions.^36^ Here we showed that the inhibition of MAPK pathway in partially reprogrammed cells affected the phosphorylation status of ERK protein in both C790 and 4434 melanoma cells independently of the de-differentiated state of the cells. Moreover, gene expression analysis of C790 reprogrammed cells revealed an upregulation of T-type calcium channels suggesting an association between the de-differentiated state of the cells and sensitivity to calcium channel antagonists. T-type calcium channels belong to a wide family of voltage gated calcium channels (VGCCs); each subfamily involves several isoforms (Cav1, Cav2 or Cav3) that display different electrophysiological properties.^37^ Channels with low voltage-activated and transient currents are known as T-type channels, which are expressed in numerous cell types including non-excitable cells.^38^ Alteration in the expression of any of the VGCCs is associated with neurological diseases, such as epilepsies,^39^ or cardiac conditions like arrhythmias.^40^ Since T-type calcium channels were reported to be upregulated in cancer cells,^41^ growing evidence has shown that VGCCs are widely expressed in many types of cancer with a particularly significant increase of T-type calcium channels in tumour cells. This overexpression has been confirmed in glioblastoma,^19,42^ ovarian cancer,^18^ breast cancer,^13^ leukaemia^17^ and melanoma.^15^ Accordingly, our results showed upregulation of T-type calcium channels in MAPKi-adaptive melanoma cells and the effect of mibefradil by inducing apoptosis and reducing the capacity of colony formation.

Previous investigations have shown that administration of T-type calcium channel antagonists (mibefradil or NNC-55–0396) inhibits proliferation and induces apoptosis in several cancer cells,^12^ i.e. U87MG glioma,^43^ colon cancer,^16^ human lung adenocarcinoma (A549), pancreatic cancer (MiaPaCa2),^14^ melanoma^44^ and leukaemia cells.^17^ Moreover, biopsies from melanoma patients show a gradual increase in T-type calcium channel expression, which relates to poor prognosis.^45^ In accordance with this, the TCGA analysis also confirmed that melanoma patients with high expression of the CACNA1H gene have poorer survival compared to patients with low expression.

Inhibition of T-type calcium channels has been used to sensitise resistant cancer cells to specific therapies or conventional chemotherapy. However, there are no previous reports of using T-type calcium channel antagonists in de-differentiated and adaptive melanoma cells in order to restore sensitivity to MAPKi. Investigations in ovarian cancer show that mibefradil enhances anti-tumour activity of carboplatin in vitro and in vivo.^18,46^ In glioblastoma, mibefradil treatment enhanced the anti-tumour effects of ionizing radiation.^47^ The combination of mibefradil with temozolomide has shown a stronger therapeutic effect and survival.^19^ Consistent with these findings our results show that mibefradil potentiates the lethal effect of MAPKi, when administered sequentially, in MAPKi-adaptive melanoma cells in vitro and in vivo. This was further supported through silencing of CACNA1H transcript in human BRAFi-adaptive melanoma cells.

The role of T-type calcium channels in de-differentiation and adaptation to therapy remains unclear. Zang et al. (2017) showed that T-type calcium channels are enriched in glioblastoma stem-like cells (GSC), which are resistant to temozolomide. They demonstrated that blockade of calcium channels can re-sensitise resistant glioblastoma cells to chemotherapy.^19^ It is known that undifferentiated mouse embryonic stem cells (ES) have voltage dependent Ca^+^ currents, which link to cell cycle progression and maintenance of self-renewal in ES cells.^48^ In a similar way, GSC show an enrichment in the expression of the Cav3.2 channel, which correlates with resistance and poor prognosis.^19^ In addition, differentiation of GSC is induced upon treatment with mibefradil, which was confirmed by downregulation of stemness markers.^19^

In order to elucidate the connection between enhanced expression of T-type calcium channels and the de-differentiation status of MAPKi-adaptive cells we evaluated specific stemness markers in murine and human melanoma cells. After single treatment with mibefradil or lomerizine, we observed a significant decrease of stemness related markers in murine de-differentiated cells. These results are in line with the reduction of *Oct3/4* and *Nanog* expression in mouse ES after pharmacological blockage of T-type calcium channels or with Cav3.2 siRNA.^48^ Consistently, adaptive resistance markers SOX2, ID1 and ID3 were also decreased in human adaptive cells after treatment with mibefradil alone or with the sequential treatment. Reduction of SOX2 expression was also confirmed with CACNA1H knockdown in human melanoma cells.^28^ Together, these results conclude that the inhibition of T-type calcium channels induces differentiation in MAPKi-adaptive cells, suggesting the participation of calcium signalling in the stemness maintenance and drug adaptation in melanoma cells.

In conclusion, we have demonstrated that inhibition of T-type calcium channels increases cell death, differentiation, and restores sensitivity of de-differentiated and adaptive melanoma cells to MAPKi. Sequential treatment with mibefradil and vemurafenib is more effective against MAPKi-adaptive melanoma cells in vitro and in vivo, reducing tumour growth and increasing overall survival in mice. Our results confirm the potential use of T-type calcium channels antagonists as an effective treatment to restore sensitivity to MAPKi in melanoma. Importantly, the safe and therapeutic use of mibefradil in humans have been evaluated in a sequential treatment with temozolomide in patients with recurrent high-grade gliomas,^49^ supporting the suitability of this new treatment approach for melanoma patients.

## Supplementary information

Supplementary tables, supplementary figures and Sup. legends

## Data Availability

All data and material presented in this article and in the supplementary information are available upon request from the corresponding author.
