# Supplementary material for: T-type calcium channel inhibition restores sensitivity to MAPK inhibitors in de-differentiated and adaptive melanoma cells
Source: Br J Cancer. 2020 Feb 17;122(7):1023–36. doi: 10.1038/s41416-020-0751-8 (PMC7109069; doi:10.1038/s41416-020-0751-8)
Supplement: Supplementary file 1 — Supplementary tables, supplementary figures and Sup. legends [file 41416_2020_751_MOESM1_ESM.pdf]

Supplementary Tables

Supplementary Table S1. List or primers (Materials and Methods section)

Mouse qPCR primers		
Amplification target	Forward Sequence	Reverse Sequence
Gapdh	AGGTCGGTGTGAACGGATTTG	TGTAGACCATGTAGTTGAGGTCA
Sox2	TTAACGCAAAAACCGTGATG	GAAGCGCCTAACGTACCACT
Ssea1	ACGGATAAGGCGCTGGTACTA	GGAAGCCATAGGGCACGAA
Mitf	CCAACAGCCCTATGGCTATGC	CTGGGCACTCACTCTCTGC
Pmel	CCTTGGGCAGGCTCCCTTGC	TCCACTGAGGAGCGGGCTGT
Cacna1h	CGGCCCTACTACGCA	ATCCTCGCTGCATTC
CD271	TGCCGATGCTCCTATGGCTA	CTGGGCACTCTTCACACACTG
Human qPCR primers		
18S	GAGGATGAGGTGGAACGTGT	TCTTCAGTCGCTCCAGGTCT
SOX2	GCCGAGTGGAAACTTTTGTCG	GGCAGCGTGTACTTATCCTTCT
ID1	CTGCTCTACGACATG	GAAGGTCCCTGATGT
ID3	GCTTGCTGGACGACA	GCGCTGTAGGATTTC
CACNA1G	GCTCCGGCACAAGTA	C CACAATGAGCAGGAA
CACNA1H	TCGAGGAGGACTTCC	TGCATCCAGGAATGG

Supplementary Table S2. IC50 values of MAPK inhibitors and calcium channel inhibitors for C790 (*Nras* mutant) during partial reprograming at days 6, 12 and 20.

C790 (Nras mutant)	IC50 values					
	Trametinib (μM)		Lomerizine (μM)		Mibefradil (μM)	
	- doxycycline	+ doxycycline	- doxycycline	+ doxycycline	- doxycycline	+ doxycycline
day 6	1.71 ± 0.99	0.20 ± 0.02	10.60 ± 0.01	5.59 ± 0.83 *	6.16 ± 0.25	3.70 ± 0.28 *
day 12	2.16 ± 1.42	> 10μM *	8.49 ± 0.16	7.18 ±0.25 *	5.75 ± 0.35	5.39 ± 0.01
day 20	2.09 ± 0.48	> 10μM **	10.02 ± 1.69	7.05 ± 0.49 *	6.44 ± 0.66	5.09 ± 0.74

Data are represented as mean±SEM of three or more independent experiments. * $p \leq 0.05$, ** $p \leq 0.01$ when compared to the control (- doxycycline) each day using unpair *t* test.

Supplementary Table S3. IC50 values of MAPK inhibitors for 4434(*Braf*^{V600E}) during partial reprograming at days 6, 12 and 20.

4434 (Braf V600E)	IC50 values					
	Trametinib (μM)		Vemurafenib (μM)		Trametinib + Vemurafenib (μM)	
	- doxycycline	+ doxycycline	- doxycycline	+ doxycycline	- doxycycline	+ doxycycline
day 6	0.0009 ± 0.0003	0.0008 ± 0.0002	5.40 ± 0.67	1.01 ± 0.05 *	0.0002 ± 0.0001	0.000007 ± 0.000001
day 12	0.0026 ± 0.0005	0.24 ± 0.06 *	4.94 ± 1.06	> 10μM **	0.0038 ± 0.0053	0.0008 ± 0.0002 **
day 20	0.0025 ± 0.0001	> 10μM ***	5.05 ± 0.35	> 10μM **	0.0029 ± 0.0020	4.38 ± 0.03 ****

Data are represented as mean ± SEM of three or more independent experiments. * $p \leq 0.05$, ** $p \leq 0.01$, *** $p \leq 0.001$, **** $p \leq 0.0001$ when compared to the control (- doxycycline) each day using unpair *t* test.

Supplementary Table S4. IC50 values of calcium channels inhibitors for 4434(*Braf*^{V600E}) during reprograming at day 20.

4434 (Braf V600E)	IC50 values			
	Mibefradil (μM)		Lomerizine (μM)	
	- doxycycline	+ doxycycline	- doxycycline	+ doxycycline
day 20	7.82 ± 0.96	8.87 ± 0.25	> 10μM	> 10μM

Data are represented as mean ± SEM of three or more independent experiments.

Supplementary Table S5. IC50 values of calcium channels inhibitor NNC 555-0396 for 4434(*Braf*^{V600E}) during reprograming at day 6, 12 and 20.

4434 (Braf V600E)	IC50 values	
	NNC 55-0396 (μM)	
	- doxycycline	+ doxycycline
day 6	6.54 ± 0.084	6.18 ± 0.059 **
day 12	6.33 ± 0.111	5.25 ± 0.018 ****
day 20	5.12 ± 0.236	3.90 ± 0.355 ***

Data are represented as mean ± SEM of three or more independent experiments. ** $p \leq 0.01$, *** $p \leq 0.001$, **** $p \leq 0.0001$ when compared to the control (- doxycycline) each day using unpair *t* test.

Supplementary Table S6. IC50 values of calcium channels inhibitor NNC 555-0396 for C790 (*Nras* mutant) during partial reprograming at days 6, 12 and 20.

C790 (Nras mutant)	IC50 values	
	NNC 55-0396 (μM)	
	- doxycycline	+ doxycycline
day 6	6.34 ± 0.916	5.82 ± 0.819
day 12	4.17 ± 0.373	3.29 ± 0.144 **
day 20	4.99 ± 0.648	3.51 ± 0.165 **

Data are represented as mean ± SEM of three or more independent experiments. ** $p \leq 0.01$ when compared to the control (- doxycycline) each day using unpair *t* test.

Supplementary Table S7. IC50 values of vemurafenib and calcium channel inhibitors for human melanoma cell lines during adaptive resistance.

Cell line	IC50 Values		
	Vemurafenib (μM)	Mibefradil (μM)	Lomerizine(μM)
A375 Parental	0.54 ± 0.58	9.63 ± 0.35	> 10 μM
A375 + Vem 24h	> 10 μM ***	7.43 ± 0.80 **	> 10 μM
SK-MEL-28 Parental	1.3 ± 0.24	8.17 ± 0.40	> 10 μM
SK-MEL-28 + Vem 24h	> 10 μM ***	6.5 ± 0.30 **	> 10 μM
HT144 Parental	0.7 ± 0.24	5.5 ± 0.41	6.24 ± 0.15
HT144 + Vem 24h	> 10 μM ***	2.65 ± 0.14 ****	3.07 ± 0.14****

Data are represented as mean ± SEM of three or more independent experiments. ** $p \leq 0.01$, *** $p \leq 0.001$, **** $p \leq 0.0001$ when compared to the control (parental) using unpair *t* test.

Supplementary Table S8. IC50 values of calcium channels inhibitor NNC 555-0396 for human melanoma cell lines during adaptive resistance.

Cell line	IC50 Values
	NNC 55-0396 (μM)
A375 Parental	5.39 ± 0.116
A375 + Vem 24h	4.63 ± 0.339 **
SK-MEL-28 Parental	6.00 ± 0.121
SK-MEL-28 + Vem 24h	5.63 ± 0.287
HT144 Parental	4.25 ± 0.034
HT144 + Vem 24h	2.77 ± 0.235 ***

Data are represented as mean ± SEM of three or more independent experiments. ** $p \leq 0.01$, *** $p \leq 0.001$ when compared to the control (parental) using unpair *t* test.

Granados *et al* Figure S1

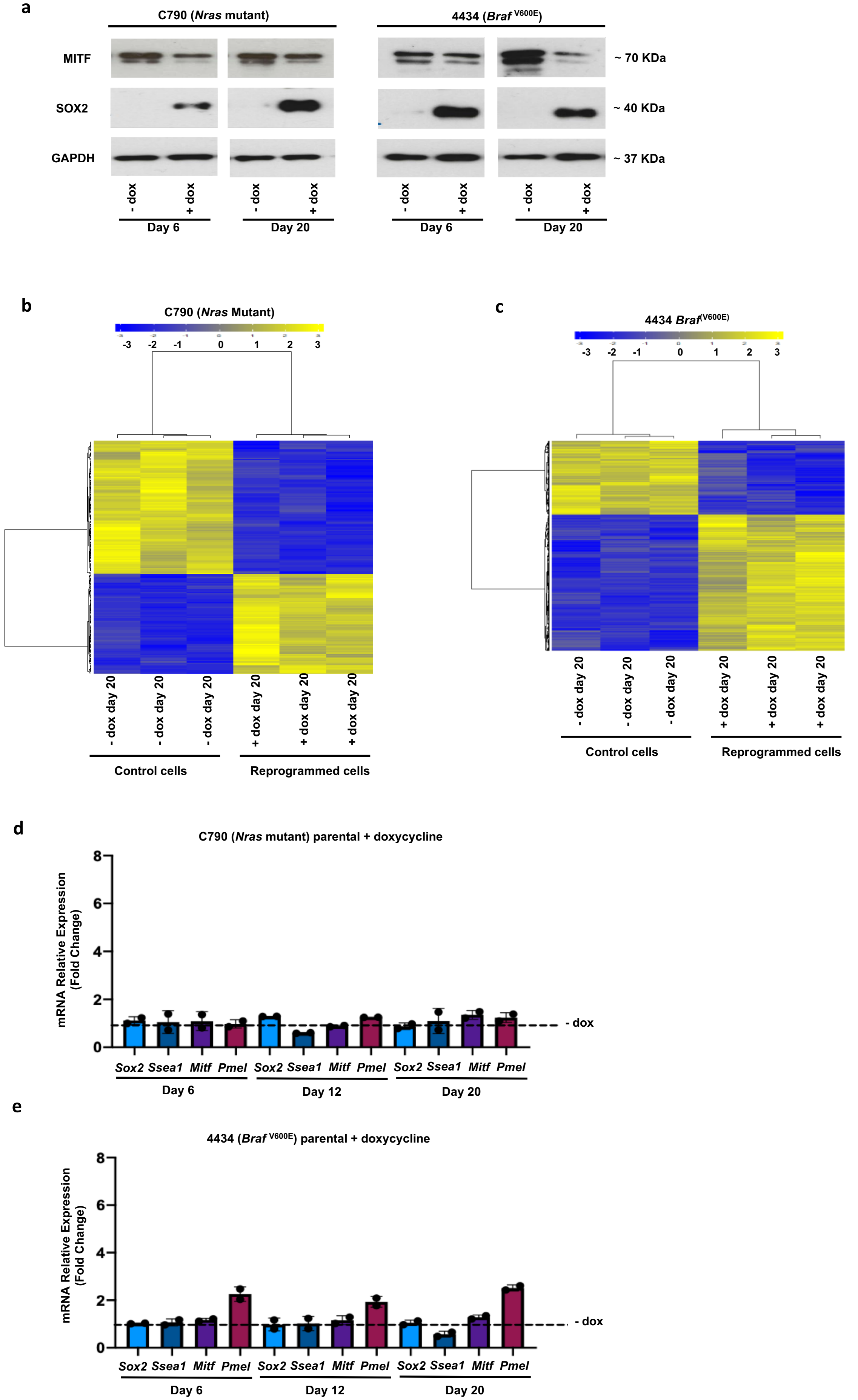

Granados *et al* Figure S2

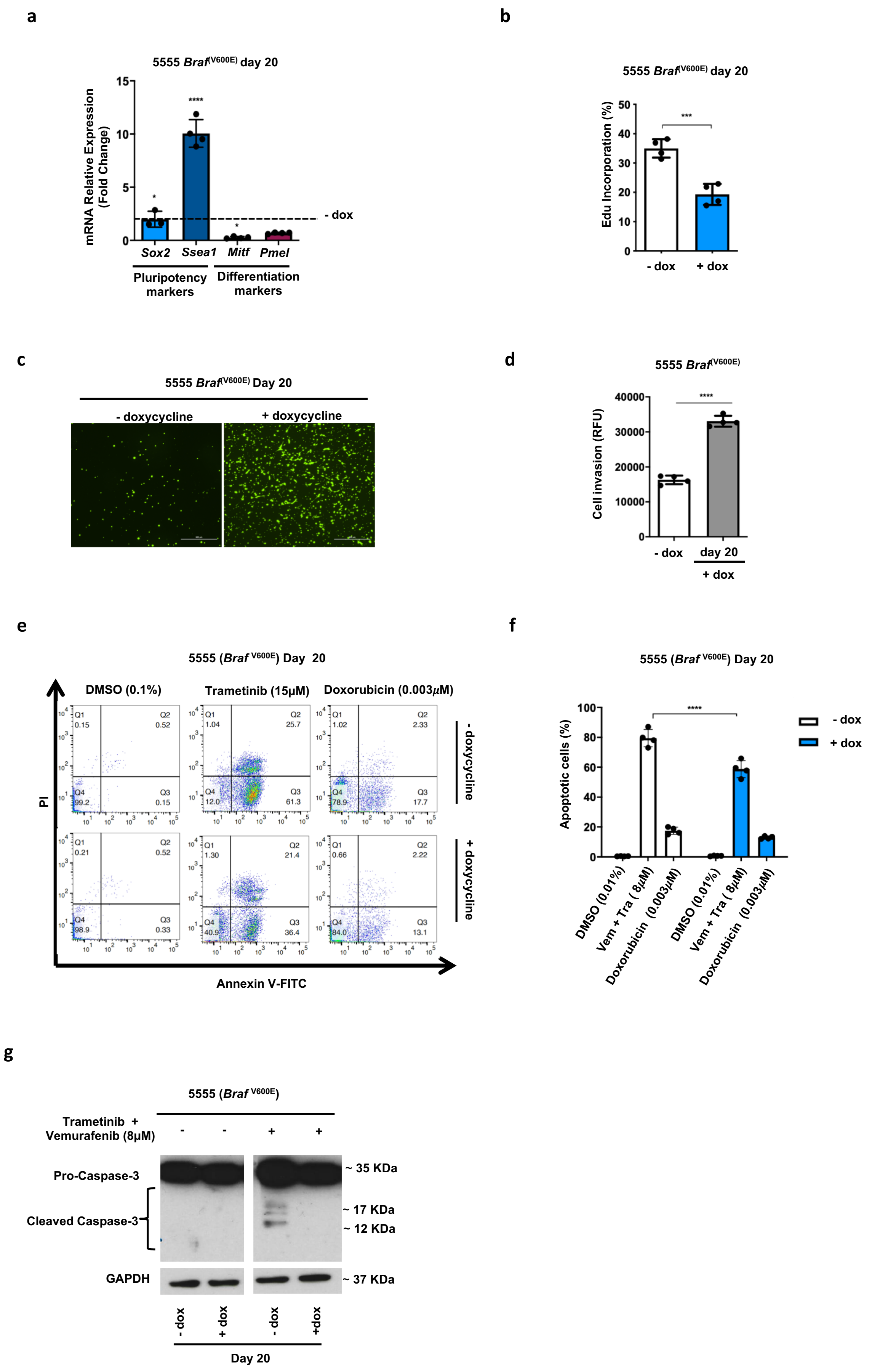

Granados *et al* Figure S3

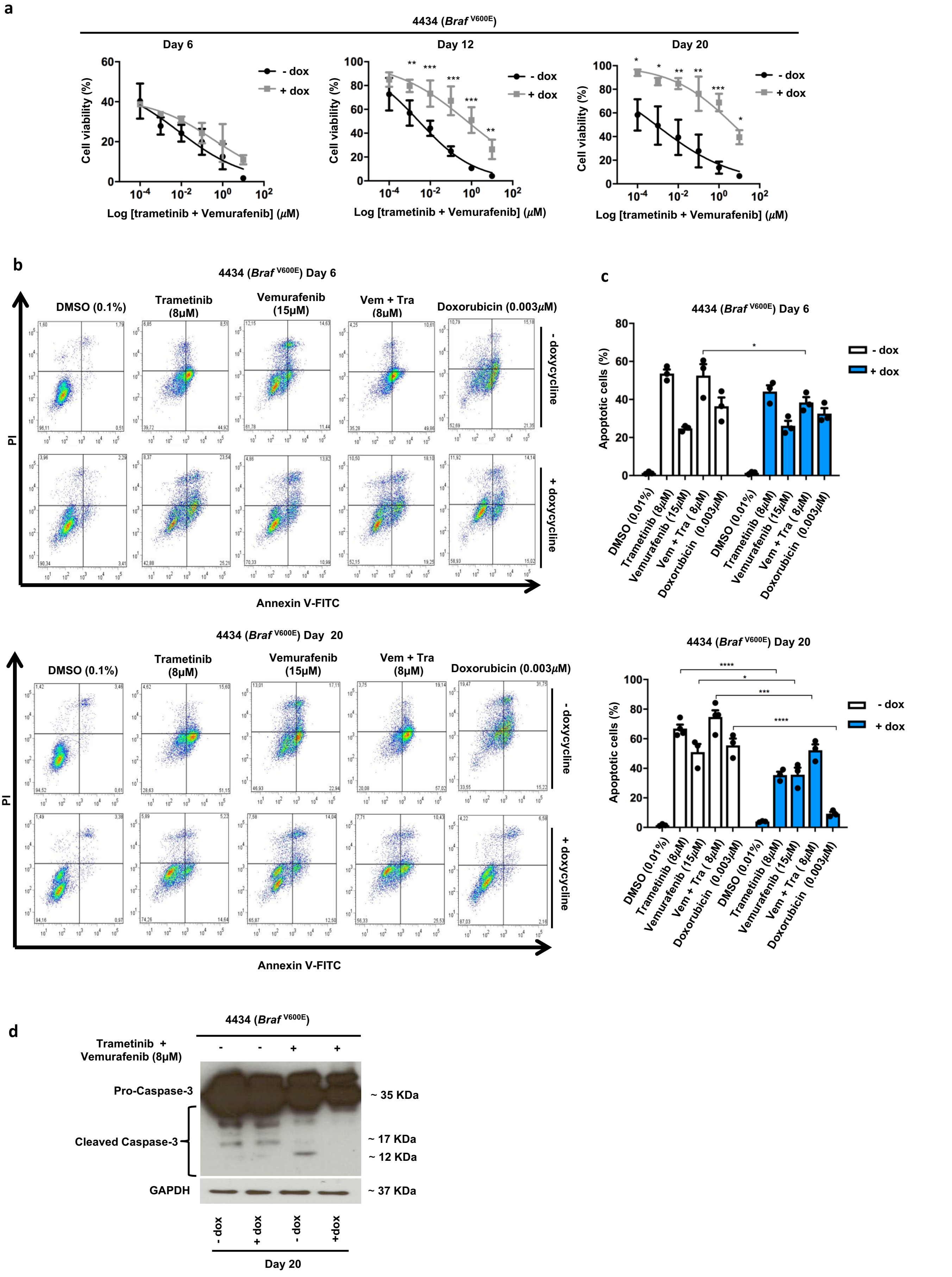

a

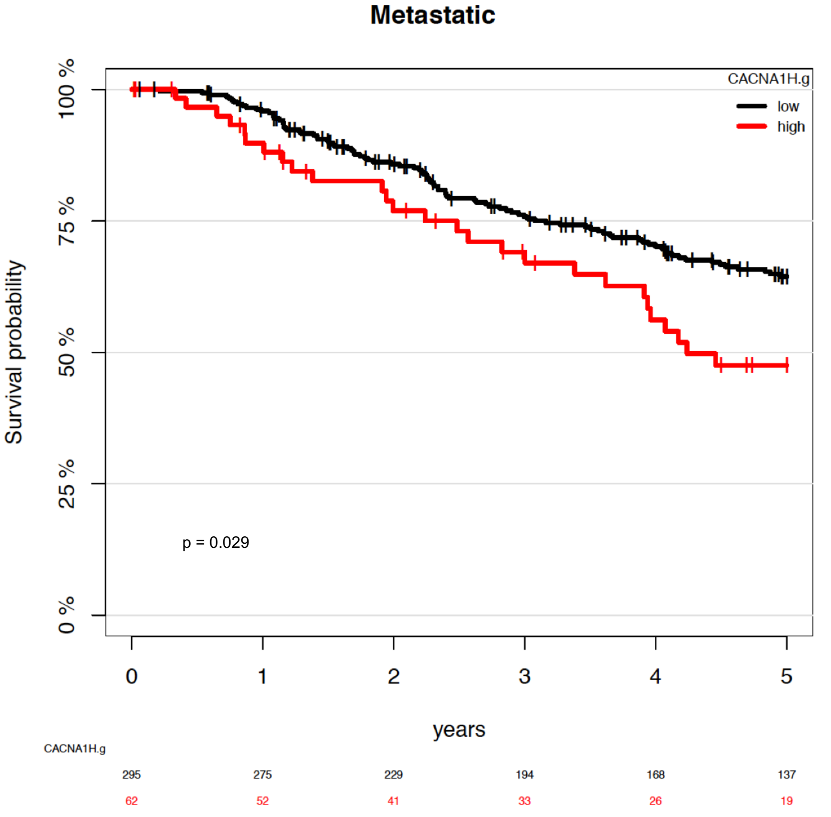
Metastatic
CACNA1H.g
low
high
Survival probability
100 %
75 %
50 %
25 %
0 %
p = 0.029
0
1
2
3
4
5
years
CACNA1H.g
295
275
229
194
168
137
62
52
41
33
26
19

b

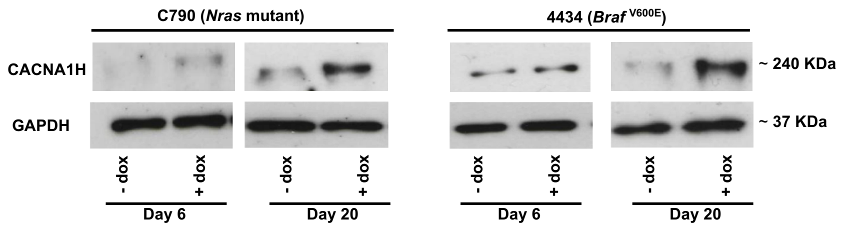
C790 (Nras mutant)
4434 (Braf V600E)
CACNA1H
GAPDH
~ 240 KDa
~ 37 KDa
- dox
+ dox
- dox
+ dox
- dox
+ dox
- dox
+ dox
Day 6
Day 20
Day 6
Day 20

Granados *et al* Figure S5

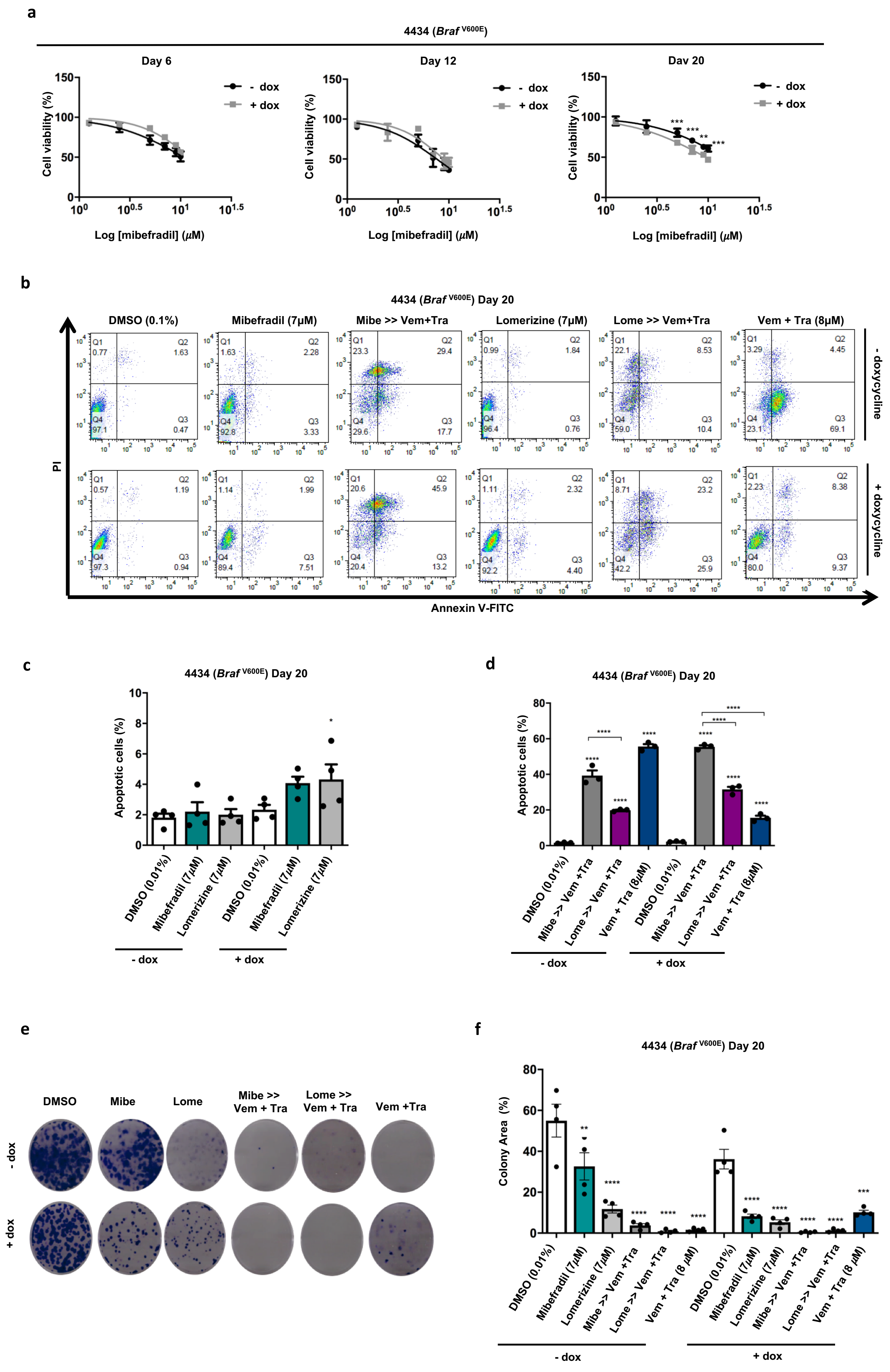

Granados *et al* Figure S6

a

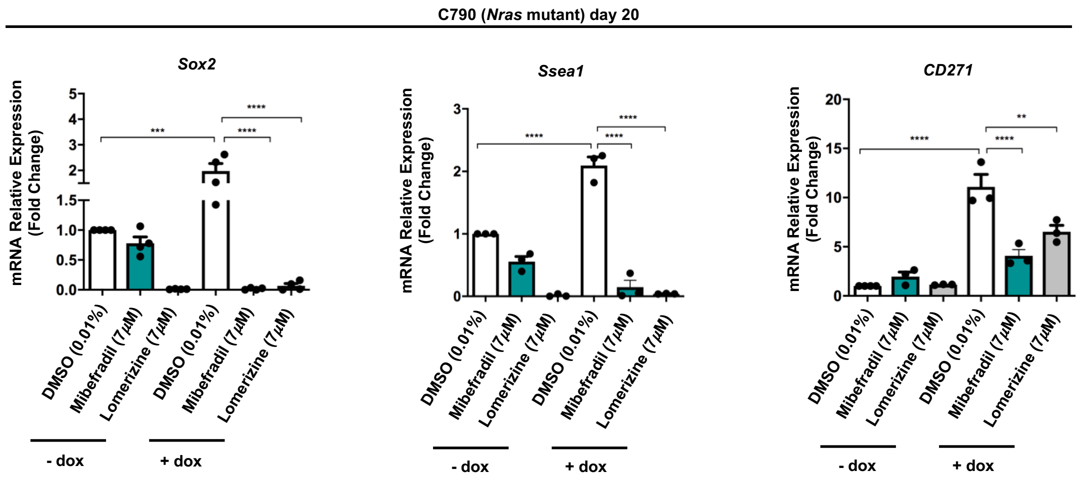

b

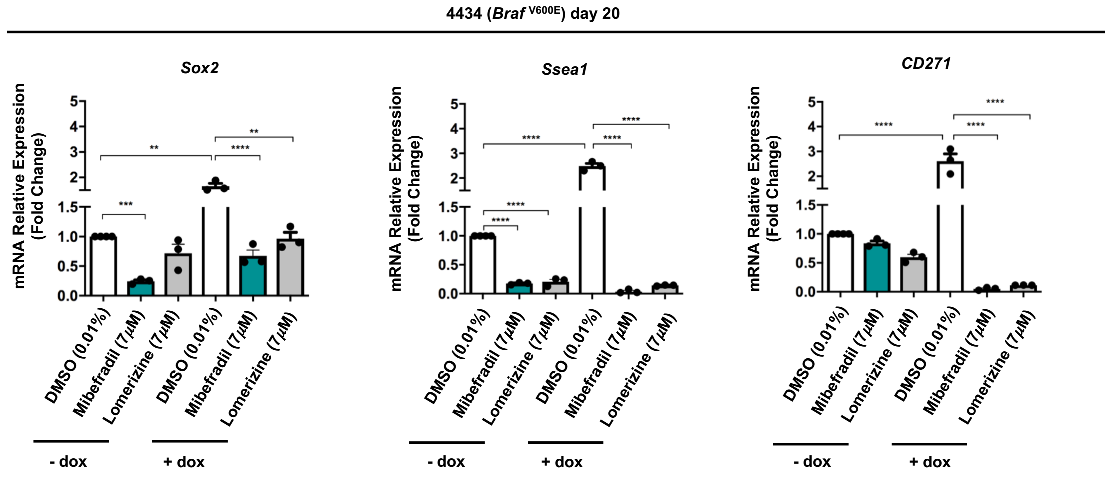

Granados *et al* Figure S7

a

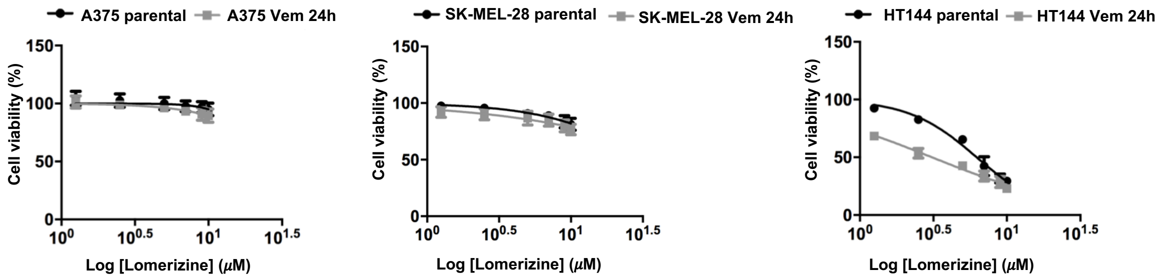

b

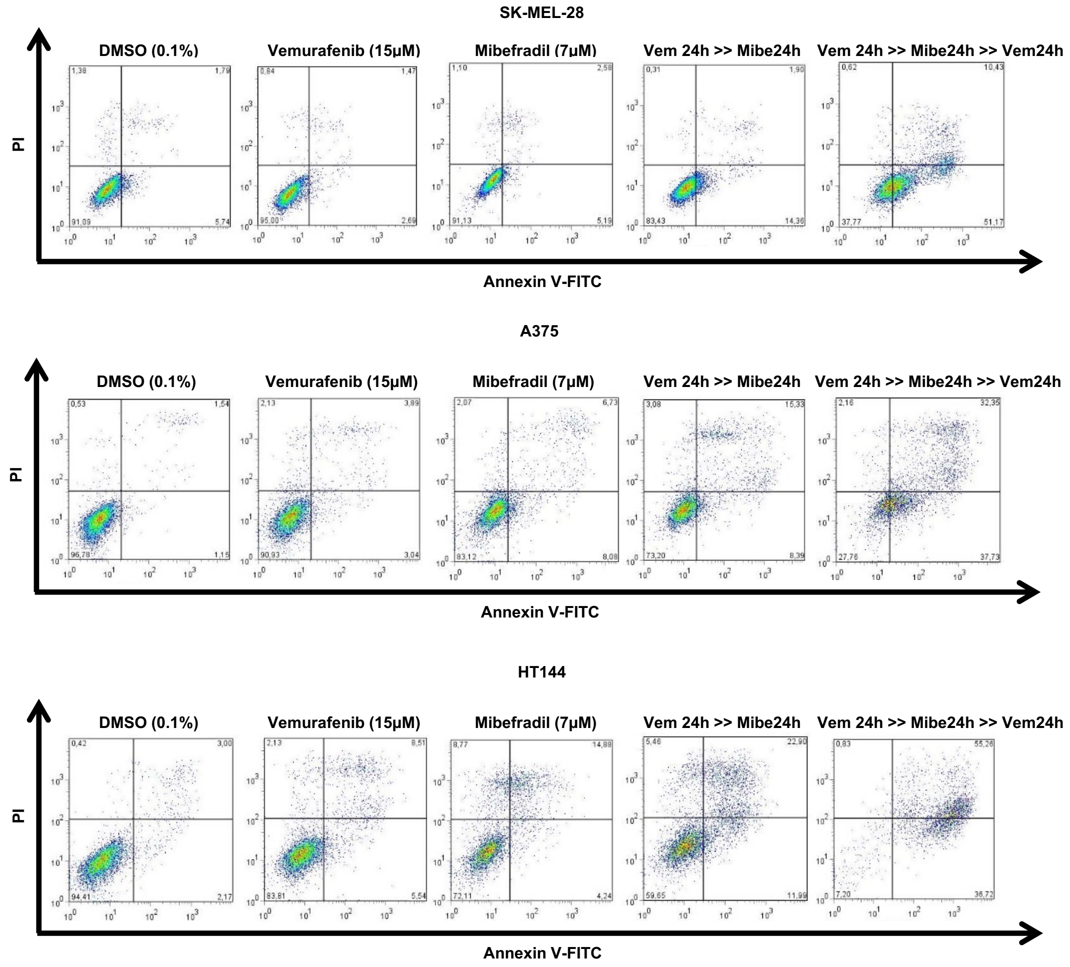

a

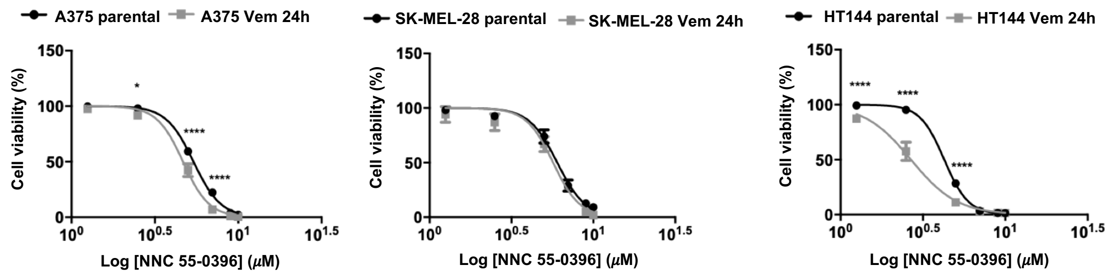
A375 parental
A375 Vem 24h
SK-MEL-28 parental
SK-MEL-28 Vem 24h
HT144 parental
HT144 Vem 24h
Cell viability (%)
Log [NNC 55-0396] (μM)
150
100
50
0
10^0
$10^{0.5}$
10^1
$10^{1.5}$
*
****

b

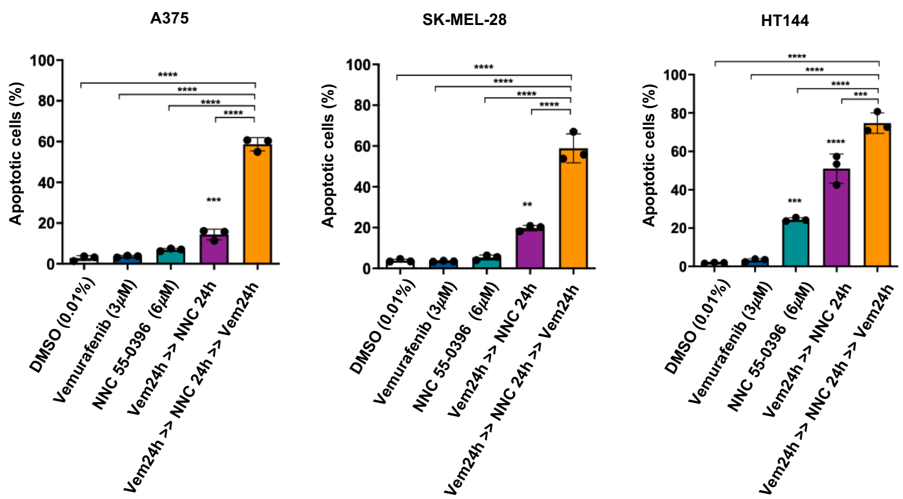
A375
SK-MEL-28
HT144
Apoptotic cells (%)
100
80
60
40
20
0
DMSO (0.01%)
Vemurafenib (3μM)
NNC 55-0396 (6μM)
Vem24h >> NNC 24h
Vem24h >> NNC 24h >> Vem24h
**
***
****

c

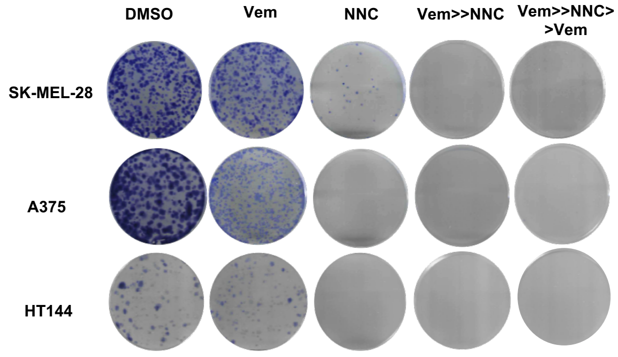
DMSO
Vem
NNC
Vem>>NNC
Vem>>NNC>
>Vem
SK-MEL-28
A375
HT144

d

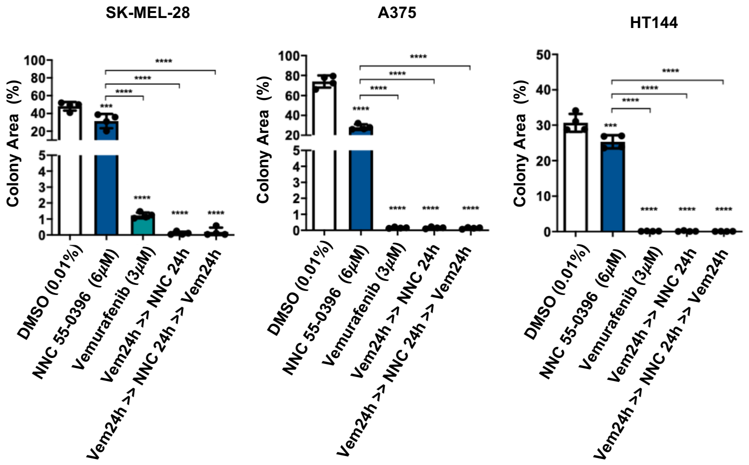
SK-MEL-28
A375
HT144
Colony Area (%)
DMSO (0.01%)
NNC 55-0396 (6μM)
Vemurafenib (3μM)
Vem24h >> NNC 24h
Vem24h >> NNC 24h >> Vem24h
***
****

Granados *et al* Figure S9

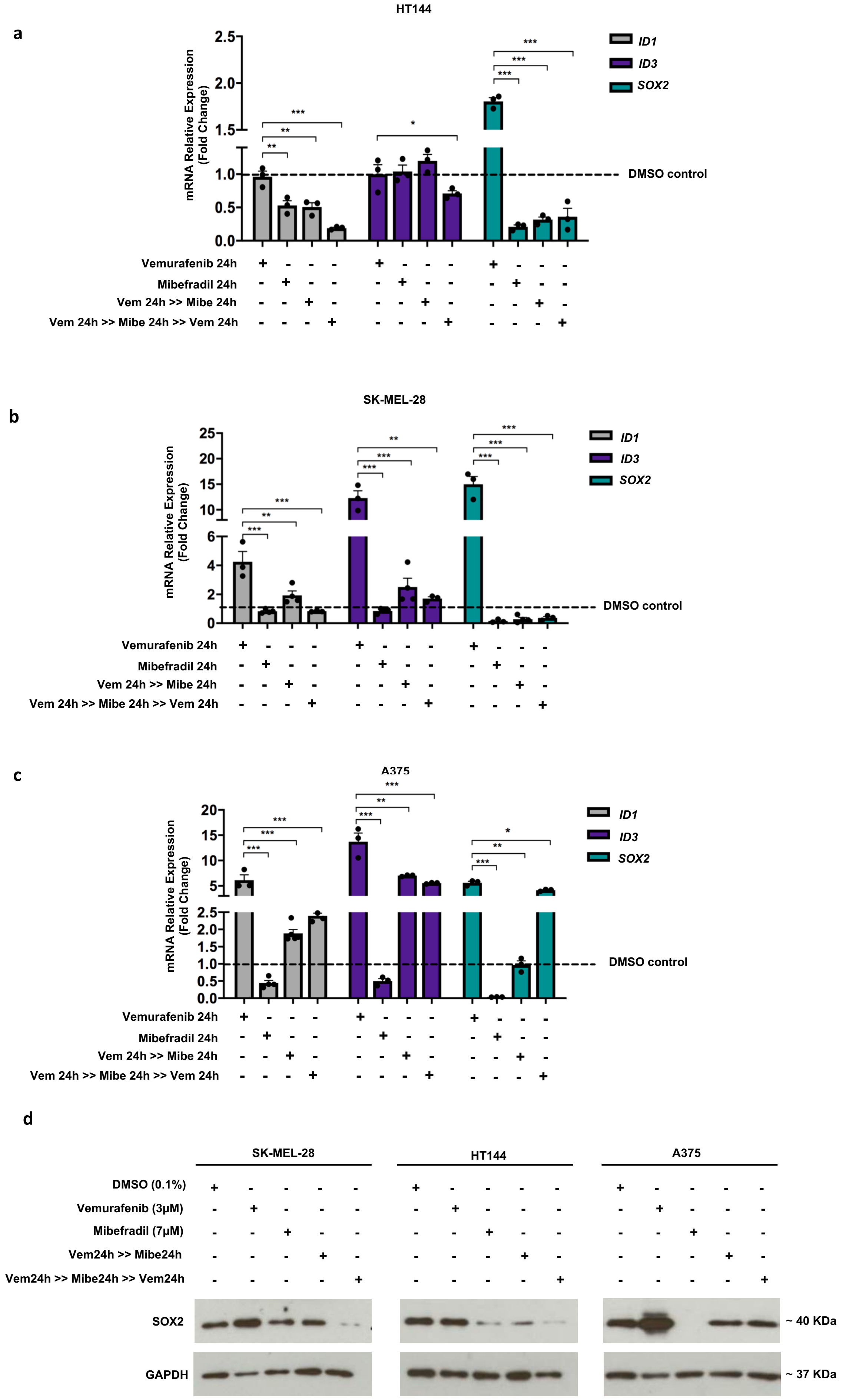

Granados *et al* Figure S10

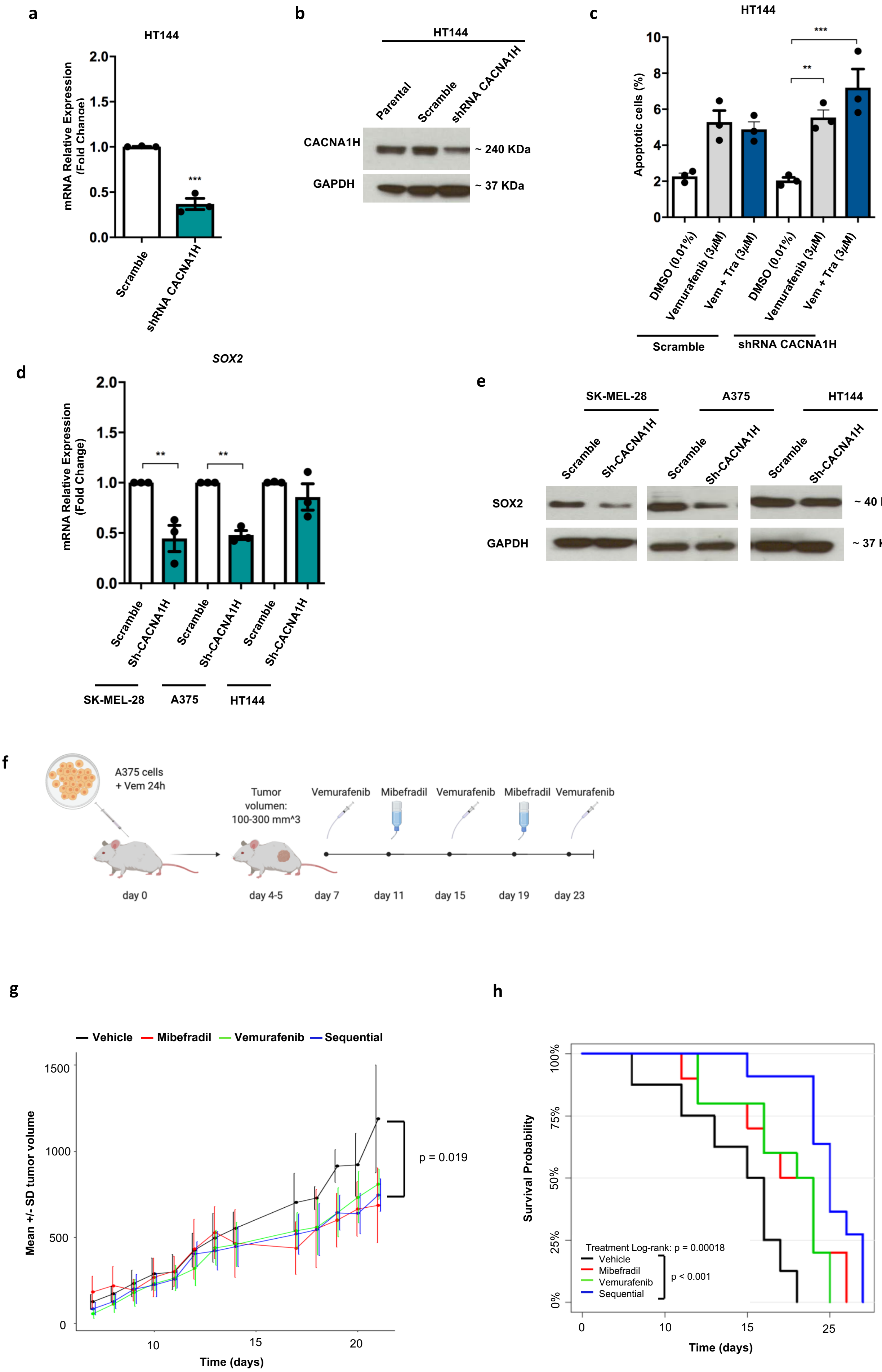

Supplementary Figures legends

Supplementary Fig. S1. Partial reprogramming of mouse cell lines decreases proliferation and differential gene expression. Related to Figure 1.

a Western Blot analysis. Whole cell lysates were immunoblotted with GAPDH, SOX2 and MITF antibodies in partially reprogrammed C790 and 4434 ("+dox") and control cells ("- dox") at day 6 and day 20. **b**, **c** Differential Gene Expression analysis. Heat map of microarray data showing hierarchical clustering of differentially expressed genes between partially reprogrammed cells C790 and 4434 ("+dox") vs control cells ("- dox") at day 20, respectively; blue or yellow colors indicate differentially up- or down regulated genes, respectively (FC > 2-fold). **d, e** Real-Time qPCR analysis for stemness markers (*Sox2* and *Ssea-1*) and melanocytic lineage differentiation markers (*Mitf* and *Pmel*) in C790 and 4434 parental cells under doxycycline treatment for over 20 days. Data were normalized using control cells ("- dox") as reference and *Gapdh* as housekeeping gene.

Supplementary Fig. S2. Partial reprogramming of 5555 mouse cell lines decreases proliferation and increase invasion. Related to Figure 1.

a Real-Time qPCR analysis for stemness markers *Sox2* and *Ssea-1*, as well as for melanocytic lineage differentiation markers *Mitf* and *Pmel* at day 20 of reprogramming. Data were normalized using control cells ("- dox") as reference and *Gapdh* as housekeeping gene. **b** Cell proliferation measured by EdU incorporation at day 20. Percentage of EdU-positive cells is shown. **c** Representative images of fluorescence microscopy for cell invasion assay in partially reprogrammed 5555 cells at day 20. Scale bars represent 500µm. **d** Cell invasion was evaluated at day 20 of reprogramming. **e** Representative scatter plots of PI (y-axis) vs. annexin V (x-axis) for day 20 of partial reprogramming. **f** Apoptosis after staining with FITC-Annexin V/PI. Cells were treated with combination of vemurafenib and trametinib, ratio 1:1 (8 µM) during 72 hours and with doxorubicin (0.003 µM) to induce apoptosis (positive control). Percentage of apoptotic cells (early and late apoptosis) is shown as mean ± SEM (n=4). **g** Western Blot analysis. Whole cell lysates were immunoblotted with GAPDH and caspase-3 antibodies at day 20, after 72 hours treatment with combination of vemurafenib and trametinib ratio 1:1 (8 µM).

Supplementary Fig. S3. 4434 partially reprogrammed cells are less sensitive to MAPKi. Related to Figure 2.

a Cell viability assay of *Braf*-mutant cells (4434). After treatment with trametinib, vemurafenib or the combination of inhibitors (10 µM to 0.0001 µM); alamar blue fluorescence emission (read at 590 nm) was obtained. Cell viability was evaluated at days 6, 12 and 20 of reprogramming. Results of combined treatment (vemurafenib + trametinib) are shown. **b** Representative scatter plots of PI (y-axis) vs. annexin V (x-axis) for days 6 and 20 of partial reprogramming. Early apoptotic cells are shown in the lower right quadrant and late apoptotic cells are shown in the upper right quadrant. **c** Apoptosis evaluation after staining with FITC-Annexin V/PI. Cells were treated with trametinib (8 µM), vemurafenib (15 µM) or the combination at ratio 1:1 (8 µM); doxorubicin (0.003 µM) was used to induce apoptosis (positive control). After 72 hours, cells were analyzed by flow cytometry. Percentage of apoptotic cells (early and late apoptosis) is shown as mean ± SEM (n=3). **d** Western Blot analysis. Whole cell lysate was immunoblotted with GAPDH and caspase-3 antibodies.

Supplementary Fig. S4. CACNA1H expression in partially reprogrammed cells and TCGA analysis of survival in melanoma patients. Related to Figure 3.

a Kaplan–Meier curves for CACNA1H expression groups on overall survival using a total of 357 metastatic melanoma patients from TCGA database. Expression values for CACNA1H gene were dichotomized into high (red) and low (black) expression using recursive partitioning ($p = 0.029$). **b** Western Blot analysis. Whole cell lysates were immunoblotted with GAPDH and CACNA1H antibodies at day 6 and 20 in partially reprogrammed C790 and 4434 cells.

Supplementary Fig. S5. Inhibition of calcium channels induces cell death in 4434 murine MAPKi-adaptive melanoma cells. Related to Figure 4.

a 4434 partially reprogrammed cells were treated with mibefradil and lomerizine (10 - 1.25 µM) for 24hours, mibefradil treatment is shown. After 3 hours of incubation with alamar blue, fluorescence emission (read at 590 nm) was obtained. Cell viability was evaluated at days 6, 12 and 20 of reprogramming. **b** Representative scatter plots of PI (y-axis) vs. annexin V (x-axis) for day 20 of partial reprogramming. Early apoptotic cells are shown in the lower right quadrant and

late apoptotic cells are shown in the upper right quadrant. **c** Apoptosis analysis. 4434 cells were treated with mibefradil (7 µM) and lomerizine (7 µM). After 24 hours, cells were analyzed by flow cytometry. Percentage of apoptotic cells (early and late apoptosis) is shown. **d** Apoptosis analysis. 4434 cells were treated sequentially with mibefradil for 24 hours, followed by vemurafenib + trametinib for another 24 hours ("Mibe >> Vem + Tra"). **e** Clonogenic assay. Colonies were stained using crystal violet. Representative images of wells stained with crystal violet are shown. **f** Clonogenic assay. 4434 cells at day 20 were treated with treatments for 24 hours. Percentage of colony area was calculated.

Supplementary Fig. S6. Inhibition of calcium channels induces differentiation in 4434 murine MAPKi-adaptive melanoma cells. Related to Figure 4.

a Real-Time qPCR analysis for stem cell markers *Sox2*, *Ssea-1* and *CD271*, at day 20 of partial reprogramming of C790 cells, after treatment with mibefradil or lomerizine. Data were normalized using control cells as reference and *Gapdh* as housekeeping gene. **b** Real-Time qPCR analysis for stem cell markers *Sox2*, *Ssea-1* and *CD271*, at day 20 of partial reprogramming of 4434 cells, after treatment with mibefradil or lomerizine. Data were normalized using control cells as reference and *Gapdh* as housekeeping gene.

Supplementary Fig. S7. Effect of mibefradil and lomerizine in human BRAF- adaptive melanoma cells. Related to Figure 5.

a Cell viability assay. After 24 hours treatment with vemurafenib, cells were treated with lomerizine (10 - 1.25 µM) for 24 hours. **b** Representative scatter plots of PI (y-axis) vs. annexin V (x-axis) for human melanoma cells after single treatment with vemurafenib (15 *µ*M), mibefradil (7 *µ*M), and sequential treatment with vemurafenib ("Vem24h>>Mibe24h>>Vem24h"). Early apoptotic cells are shown in the lower right quadrant and late apoptotic cells are shown in the upper right quadrant.

Supplementary Fig. S8. NNC 55-0396 increases vulnerability to MAPK inhibitors in human BRAF-adaptive melanoma cells.

a Human melanoma cells were treated with NNC 55-0396 (10,9,7,5,2.5 and 1.25 µM) for 24 hours. After 3 hours of incubation with alamar blue, fluorescence (590 nm) was obtained. **b** Apoptosis analysis, human melanoma cells were treated with vemurafenib (3 µM) for 24 hours, followed by NNC 55-0396 (6 µM) for 24 hours; after this period cells were re-treated with vemurafenib (3µM) for another 24 hours ("Vem 24h >> NNC 24h >> Vem 24h"). Cells were stained and analyzed by flow cytometry. Percentage of apoptotic cells (early and late apoptosis) is shown as mean ± SEM (n=3). **c** Clonogenic assay of human cells A375, SK-MEL-28 and HT144 treated for 24 hours with DMSO (0.01%), NNC 55-0396 (6 µM), vemurafenib (3 µM) and sequential treatment (Vem 24h >> NNC 24h >> Vem 24h). Representative images of wells stained with crystal violet are shown. **d** Percentage of colony area for all treatments is shown as mean ± SEM (n=3) in all human melanoma cell lines.

Supplementary Fig. S9. Mibefradil induces differentiation in human BRAF-adaptive melanoma cells. Related to Figure 5.

a,b,c qPCR analysis of expression of adaptive resistance markers *SOX2*, *ID1* and *ID3* in SK-MEL-28, A375 and HT144, after treatment with mibefradil alone or sequential treatment with vemurafenib ("Vem24h>>Mibe24h>>Vem24h"). Data were normalized using control cells as reference and 18S as housekeeping gene. **d** Western Blot analysis. Whole cell lysate was immunoblotted with GAPDH and SOX2 antibodies.

Supplementary Fig. S10. Silencing of CACNA1H in human cell lines and inhibition of tumor growth *in vivo* in A375 human BRAFi-adaptive melanoma xenografts. Related to Figure 6.

a qPCR analysis of CACNA1H after knockdown with shRNA CACNA1H in HT144 cell line. **b** Western Blot analysis of CACNA1H after knockdown with shRNA CACNA1H in HT144 cell line. **c** Apoptosis analysis. Percentage of apoptotic cells after treatment with combination of vemurafenib and trametinib in HT144- CACNA1H knockout cells. **d** qPCR analysis of SOX2 after silencing CACNA1H in human cells: SK-MEL-28, A375 and HT144. Data were normalized using control cells (scramble) as reference and 18S as housekeeping gene. **e** Western Blot analysis. Whole cell lysates of CACNA1H -knockdown human cells: SK-MEL-28, A375 and HT144, were

immunoblotted with GAPDH and SOX2 antibodies. **f** Workflow of *in vivo* evaluation of sequential treatment (Vem >> Mibe >> Vem) in human adaptive cells. A375 cells treated with vemurafenib for 24 hours were injected subcutaneously in female NGS mice. Once tumor volume reached 100-300 mm^3, animals were randomly divided into 4 groups (n=12), and treatments were administered daily as depicted. **g** Effect of sequential treatment on tumor growth in A375 xenografts, shown as mean and standard deviation of tumor volume over time following treatment initiation. P-values were adjusted for multiple testing. **h** Kaplan-Meier curves representing survival of A375 xenografts mice treated with vehicle (black line), mibefradil (red line), vemurafenib (green line) and sequential treatment (blue line). The survival curves were analyzed with pairwise treatment comparison using log-rank test with adjustment of p-values for multiple testing.
